# Haplotype‐resolved telomere‐to‐telomere genome of the jade vine (*Strongylodon macrobotrys*) provides novel insights into the turquoise flower coloration

**DOI:** 10.1111/jipb.70136

**Published:** 2026-01-20

**Authors:** Tong‐Jian Liu, Xin‐Feng Wang, Ding‐Ding Shi, Zhi‐Qiang Wang, Gui‐Qi Bi, Zhe‐Li Lin, Hui‐Run Huang, Xue‐Jun Ge, Lin‐Feng Li, Hai‐Fei Yan, Shao‐Hua Zeng, Zu‐Lin Ning

**Affiliations:** ^1^ Guangdong Provincial Key Laboratory of Applied Botany South China Botanical Garden, The Chinese Academy of Sciences Guangzhou 510650 China; ^2^ Key Laboratory of National Forestry and Grassland Administration on Plant Conservation and Utilization in Southern China South China Botanical Garden, The Chinese Academy of Sciences Guangzhou 510650 China; ^3^ Coconut Research Institute of Chinese Academy of Tropical Agricultural Sciences Wenchang 571339 China; ^4^ School of Biology and Agriculture Shaoguan University Shaoguan 512005 China; ^5^ School of Life Sciences Sun Yat‐sen University Guangzhou 510275 China; ^6^ University of the Chinese Academy of Sciences Beijing 100049 China

## Abstract

A haplotype‐resolved telomere‐to‐telomere genome reveals that the bird‐shaped turquoise flowers of *Strongylodon macrobotrys* (jade vine) arise from co‐pigmentation between the anthocyanin malvin and the flavonoid saponarin, shaped by genome dynamics and geological event‐associated expansions of long terminal repeat retrotransposons

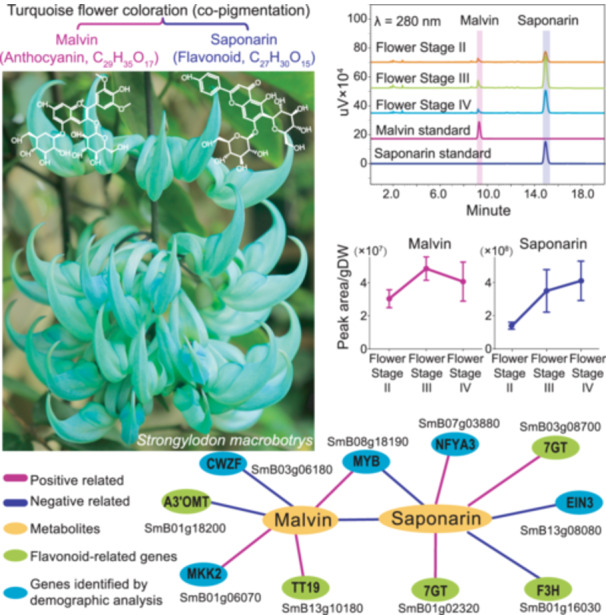

The origin and diversification of flowers are key evolutionary innovations in angiosperms, profoundly shaping biodiversity and ecosystem function (Specht and Bartlett, 2009; Soltis et al., 2018). Floral traits like color, shape, and scent promote plant diversification and co‐evolution with pollinators (Narbona et al., 2021). Flower color shows remarkable diversity, driven by complex biochemical pathways and selective pressures (Narbona et al., 2021). While common colors like white, yellow, red, and purple dominate globally, turquoise hues are rare and genetically understudied, especially in non‐model plants (Narbona et al., 2021). *Strongylodon macrobotrys* A. Gray, or jade vine, a rare climbing rainforest legume endemic to the Philippines, is known for its luminous turquoise flowers that attract birds and bats (Figure 1A). Like other legumes, *S. macrobotrys* has the ability to fix atmospheric nitrogen, thereby contributing significantly to ecosystem nutrient cycling (Sprent, 2009; Liu et al., 2024). It is now threatened by limited distribution and habitat loss (Andrews and Lewis, 1984; Konishi, 1999). Previous studies linked its unique color to co‐pigmentation between the anthocyanin Malvin (malvidin‐3,5‐O‐diglucoside) and flavonoid co‐pigment Saponarin (isovitexin‐7‐O‐glucoside) under mildly alkaline conditions (Takeda et al., 2010), but the molecular basis remains unknown. In this study, we generated a high‐quality genome and integrate transcriptomic and metabolomic data to uncover the genetic and evolutionary basis of turquoise flower pigmentation, providing new insights into flower color innovation in tropical plants.

**Figure 1:**
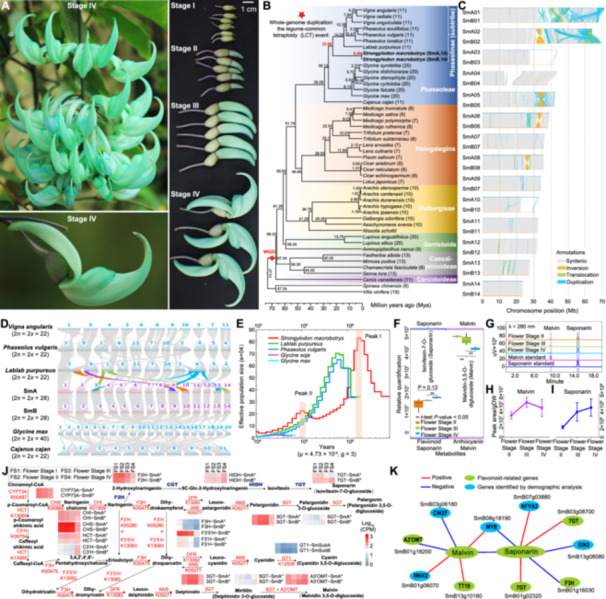
**Genome characterization and evolutionary analysis of** ***Strongylodon macrobotrys*** **(A)** Inflorescences, flowers, and buds of *S. macrobotrys*, showing four flowering stages: Stage I (bud initiation), stage II (bud enlargement), stage III (early flowering), and stage IV (full bloom). **(B)** Phylogeny and divergence times of Fabaceae species, with basic chromosome numbers shown in parentheses. **(C)** Structure variants between the two *S. macrobotrys* haplotype genomes. **(D)** Genome synteny between *S. macrobotrys* haplotypes and five other Phaseoleae species. **(E)** Demographic history inferred by PSMC. **(F)** Boxplot of Malvin and Saponarin levels across flowering stages II–IV based on metabolomic analysis. Differences among stages were assessed using a two‐tailed Student's *t*‐test (*P*‐value < 0.05). **(G)** High‐performance liquid chromatography (HPLC) profiles of Malvin and Saponarin at flowering stages II–IV, detected at 280 nm. **(H**–**I)** Quantification of Malvin and Saponarin across flowering stages II–IV, expressed as chromatographic peak area per unit dry weight (means ± *SD*, *n* = 3). **(J)** Predicted biosynthetic pathways of anthocyanins and Saponarin. Adjacent heatmaps show the summed expression of all gene copies encoding key enzymes across flowering stages I–IV in each haplotype genome. Asterisks indicate gene copies differentially expressed across stages. **(K)** Metabolite–gene network of Malvin and Saponarin highlighting associated biosynthetic and demography‐related genes.

Here, we present a haplotype‐resolved, telomere‐to‐telomere (T2T) genome assembly of *S. macrobotrys*, utilizing 70.04 Gb (~120×) of PacBio HiFi reads, 32.4 Gb (~56×) of ONT ultra‐long reads, and 233.6 Gb (~403×) of Hi‐C data (Table S1). This strategy produced two haplotype assemblies, SmA and SmB, with total sizes of 604.68 Mb and 686.34 Mb and contig N50 values of 23.99 Mb and 25.31 Mb, respectively (Figures S1–S5; Tables S1–S6). Over 94% of both assemblies were anchored to 14 pseudo‐chromosomes. We predicted 31,898 and 34,241 protein‐coding genes in SmA and SmB (Tables S6–S10). High mapping efficiencies were observed, exceeding 97.88% for whole‐genome reads and 99.95% for HiFi reads. BUSCO scores were 98.4% (SmA) and 98.6% (SmB), with Merqury high‐quality values of 59.71 and 61.90 (Table S6). Centromeres were detected across all 28 pseudo‐chromosomes, and telomeres were identified at both ends of 13 SmA and 12 SmB pseudo‐chromosomes and at one end of three others (Figure S5; Tables S11, S12). Repetitive sequences accounted for 62.39% (SmA) and 58.94% (SmB), including multiple LTR (Long terminal repeat retrotransposons) bursts (Figure S6; Tables S6, S13). Two *Gypsy*‐driven waves and a recent *Copia* burst highlight elevated and dynamic transposable element (TE) activity (Figure S6).

Phylogenomic analysis of 45 Fabaceae genomes positioned *S. macrobotrys* at a basal lineage of the subtribe Phaseolinae, sister to *Vigna*, *Phaseolus*, and *Lablab*, diverging ~22.9 million years ago (Mya) (Figure 1B; Tables S14–S19). The two *S. macrobotrys* haplotypes diverged around 0.49 Mya, indicating substantial genomic differentiation. Extensive structural variations (non‐collinear) between SmA and SmB affected about 15%–17% of the genome, with prominent inversions on pseudo‐chromosomes 02, 05, 06, and 08, alongside widespread duplications and translocations across nearly all pseudo‐chromosomes (Figure 1C; Tables S14–S17). Within the tribe Phaseoleae, *S. macrobotrys* and related species show notable variation in basic chromosome numbers, ranging from *x* = 11 to 20 (Figure 1B; Table S18). Notably, *S. macrobotrys* uniquely possesses a base number of 14. Synteny analysis suggests that this chromosomal diversity results mainly from multiple fission, fusion, and rearrangement events, as illustrated by pseudo‐chromosomes 03, 09, 10, 13, and 14 compared with relatives, reflecting complex karyotype evolution (Figure 1D). Genome‐wide synteny and synonymous substitution rates (Ks) confirm the ancient legume‐common tetraploidy (LCT) event, with no evidence of recent whole‐genome duplications (Figure S7).

To explore the population history of *S. macrobotrys*, we applied the pairwise sequentially Markovian coalescent (PSMC) model to infer changes in effective population size (Ne) over time. Two expansion events were detected (Figure 1E): Peak I (~2.5 Mya) during the Pliocene–Pleistocene transition and Peak II (~0.15 Mya), coinciding with the Penultimate Glacial Maximum (PGM) and potential colonization of exposed continental shelves (Raes et al., 2014). These demographic shifts of *S. macrobotrys* differed markedly from related species (Figure 1E), reflecting unique evolutionary dynamics of the Southeast Asian tropical rainforests. Ks‐based analysis identified 48 and 436 genes associated with Peaks I and II, respectively (Tables S20, S21). Peak I genes were enriched in gene regulation, while Peak II genes were involved in stress responses, flavonoid metabolism, and blue‐light signaling—key to anthocyanin biosynthesis (Table S21). Notably, LTRs were found near most peak‐associated genes (91.67% in Peak I and 80.05% in Peak II), suggesting that TEs may have contributed to adaptation during environmental changes.

The turquoise floral color of *S. macrobotrys* is a striking ornamental trait, previously linked to co‐pigmentation between Malvin and Saponarin (Takeda et al., 2010). However, the molecular basis of this pigmentation remains unclear. To address this, we conducted integrated multi‐omics analysis during floral development (Figures 1F–J, S8–S23; Tables S22–S34). Metabolomics showed a general decline in anthocyanins during floral development, particularly at the final flowering stage (Stage IV), across all three biosynthetic branches (delphinidin, cyanidin, and pelargonidin) (Figures 1F, S8; Tables S22, S23). In contrast, Saponarin levels increased, consistent with its role in stabilizing turquoise color despite being colorless or pale yellow (Takeda et al., 2010). Among the anthocyanins, delphinidin‐derived compounds, typically associated with blue to blue–purple pigmentation, were predominant, with Malvin being the most abundant (Figures 1F, S8). Chromatographic and mass spectrometric analyses confirmed that Saponarin levels progressively increased during floral development, whereas Malvin showed fluctuations and ultimately declined at flowering stage IV (Figures 1G–I, S9). In contrast, cyanidin (purple–red) and pelargonidin (orange–red) derivatives were significantly reduced, with pelargonidins nearly undetectable at Stage IV (Figure S8).

Gene expression analyses identified coordinated downregulation of key anthocyanin biosynthetic genes (e.g., *CHS*, *F3′H*, *F3′5′H*, and *3GT*) and upregulation of Saponarin biosynthetic gene (*HIDH*) at stage IV (Figures 1J, S10–S27; Tables S24–S34). Co‐expression network analysis revealed that *A3′OMT* and *TT19* were coexpressed with Malvin, while *F3H* and *7GT* were coexpressed with Saponarin (Figure 1K). Several genes (*MYB*, *EIN3*, and *MKK2*), associated with demographic expansion (Peak II) and nearby LTRs, were also coexpressed with flavonoid pathway genes. Their homologs directly or indirectly regulate anthocyanin biosynthesis in other species, suggesting that demographic expansion and LTR activity may have contributed toward shaping turquoise floral pigmentation (Figure 1K). Quantitative real‐time polymerase chain reaction (qRT‐PCR) validation of these key genes (e.g., *A3′OMT*, *F3H*, *7GT*, *MYB*, *EIN3*, and *MKK2*) confirmed expression patterns consistent with RNA‐seq data (Figure S28; Table S35). Together, these findings show how coordinated shifts in pigment biosynthesis and transcriptional regulation shaped the emergence of this rare floral trait. Our study reveals the genetic basis of turquoise coloration and provides a valuable genomic resource for investigating floral innovation and adaptation in legumes and tropical plants.

## CONFLICTS OF INTEREST

The authors declare no conflicts of interest.

## AUTHOR CONTRIBUTIONS

Z.L.N., S.H.Z., and H.F.Y. designed and supervised the research; T.J.L., X.F.W., and D.D.S. analyzed the data and wrote the manuscript; and other authors contributed to data analysis. All authors have read and approved the contents of this paper.

## Supporting information

Additional Supporting Information may be found online in the supporting information tab for this article: http://onlinelibrary.wiley.com/doi/10.1111/jipb.70136/suppinfo

**Figure S1.** Photograph of a chromosome spread from the root tip cells of *Strongylodon macrobotrys*, with each of the 28 chromosomes clearly labeled for counting and identification
**Figure S2.** Flow cytometry estimation of *S. macrobotrys* genome size, using *Oryza sativa* as an internal standard for comparison
**Figure S3.** K‐mer spectrum analyses (*k* = 19 and *k* = 21) for the *S. macrobotrys* genome survey
**Figure S4.** Two‐dimensional heatmaps generated using Smudgeplot (*k* = 21) illustrate the predicted ploidy of *S. macrobotrys*
**Figure S5.** Circos plot of the haplotype‐resolved T2T genome assembly of *S. macrobotrys*
**Figure S6.** Transposable element dynamics in *S. macrobotrys*
**Figure S7.** Synonymous substitution rate (Ks) distributions reveal whole‐genome duplication and divergence history
**Figure S8.** Boxplot of anthocyanin and saponarin levels across flowering stages (Stage II–IV) based on metabolomic analysis
**Figure S9.** Negative ion mass spectrum of malvin and saponarin
**Figure S10.** UpSet plot summarizing the intersections of expressed genes (CPM > 1) across all tissues77
**Figure S11.** UpSet plot summarizing the intersections of lowly expressed genes (CPM ≤ 1) across all tissues
**Figure S12.** UpSet plot summarizing the intersections of non‐expressed genes across all tissues
**Figure S13.** Heatmap of gene expression profiles across different tissues
**Figure S14.** Heatmap of gene expression profiles across different tissues (with clustering)
**Figure S15.** Volcano plots showing differentially expressed genes (DEGs) between flower tissues at developmental stages I–IV (FS1–FS4).
**Figure S16.** Summary of downregulated genes (downDEGs) in flower tissues at developmental stages I–IV (abbreviated as FS1–FS4) compared with leaf, root, and stem tissues
**Figure S17.** Summary of upregulated genes (upDEGs) in flower tissues at developmental stages I–IV (FS1–FS4) compared with leaf, root, and stem tissues
**Figure S18.** Significantly enriched KEGG (ko) terms among downDEGs in flower tissues at developmental stages I–IV (FS1–FS4), identified from pairwise comparisons indicated along the X‐axis
**Figure S19.** Significantly enriched KEGG (ko) terms among upDEGs in flower tissues at developmental stages I–IV (FS1–FS4), identified from pairwise comparisons indicated along the X‐axis
**Figure S20.** Significantly enriched KEGG (ko) terms among shared downDEGs and upDEGs in flower tissues at developmental stages I–IV (FS1–FS4), identified from pairwise comparisons indicated along the X‐axis
**Figure S21.** UpSet plot summarizing the intersection of downDEGs (a) and upDEGs (b) across all pairwise comparisons among flower tissues at different developmental stages (FS1–FS4)
**Figure S22.** Significantly enriched KEGG (ko) terms among upDEGs during flower development, identified from pairwise comparisons indicated along the X‐axis
**Figure S23.** Significantly enriched KEGG (ko) terms among upDEGs during flower development, identified from pairwise comparisons indicated along the X‐axis
**Figure S24.** Significantly enriched KEGG (ko) terms among shared downDEGs and upDEGs during flower development, identified from pairwise comparisons indicated along the X‐axis
**Figure S25.** Heatmaps showing gene expression profiles across various tissues.
**Figure S26.** Heatmap of the expression levels of five enzyme genes involved in the anthocyanin and flavone biosynthetic pathways
**Figure S27.** Heatmap of the cumulative expression levels of all same gene copies encoding enzymes associated with anthocyanin and flavonoid biosynthetic pathways of two *S. macrobotrys* haplotype genomes: SmA and SmB
**Figure S28.** Quantitative real‐time polymerase chain reaction (qRT‐PCR) analysis performed to confirm the transcriptomic data

**Table S1.** Sequencing information of genome survey, assembly, scaffolding, and annotation for *Strongylodon macrobotrys*
**Table S2.** Flow cytometry estimates of *S. macrobotrys* genome size compared to internal standards of *Oryza sativa* (japonica group; NCBI accession: GCF_001433935.1)
**Table S3.** Ploidy estimation of *S. macrobotrys* by nQuire using whole genome sequencing data.
**Table S4.** Estimation of the ploidy level of *S. macrobotrys* in Smudgeplot
**Table S5.** Genome profiling of *S. macrobotrys* inferred by Genomescope2 using the diploid model.
**Table S6.** Statistics for the *S. macrobotrys* haplotype‐resolved genome assembly
**Table S7.** Telomere repeats (AAACCCT) identified on 28 pseudo‐chromosomes of *S. macrobotrys* genome
**Table S8.** Centromere repeats identified on 28 pseudo‐chromosomes of the *S. macrobotrys* genome
**Table S9.** Protein‐coding genes predicted by braker2 pipeline in the *S. macrobotrys* genome
**Table S10.** Functional prediction of proteins harbored from two *S. macrobotrys* haplotype genomes
**Table S11.** Copy numbers of nuclear ribosomal RNAs identified in the *S. macrobotrys* genome
**Table S12.** Copy numbers of non‐coding RNAs identified in the *S. macrobotrys* genome
**Table S13.** Summary of repeat sequence annotations for *S. macrobotrys* using Earlgrey
**Table S14.** Structure divergence between two *S. macrobotrys* haplotype genomes using SyRI
**Table S15.** Information of syntenic blocks for *S. macrobotrys* haplotype genome A (SmA) using WGDI
**Table S16.** Information of syntenic blocks for *S. macrobotrys* haplotype genome B (SmB) using WGDI
**Table S17.** Information of syntenic blocks between two *S. macrobotrys* haplotype genomes using WGDI
**Table S18.** Detailed information of 43 Fabaceae genomes and two outgroups used in comparative genomics and phylogenomic analyses
**Table S19.** The seven fossil calibrations imposed by divergence time estimation.
**Table S20.** Gene functions for Peak I in the demographic analysis of *S. macrobotrys* genomes using PSMC.
**Table S21.** Gene functions for Peak II in the demographic analysis of *S. macrobotrys* genomes using PSMC.
**Table S22.** Metabolite profiles during floral developmental stages II–IV (referred to as FS2–FS4) of *S. macrobotrys*
**Table S23.** Relative metabolomic abundance of anthocyanins and saponarin across different flower samples.
**Table S24.** Genome‐wide gene expression matrix (CPM values) of flower and other tissues in *S. macrobotrys*
**Table S25.** Differentially expressed gene (DEG) matrix across floral stages and other tissues in *S. macrobotrys* (1 = significantly downregulated, 2 = significantly upregulated, and 0 = not significant)
**Table S26.** Summary of genes expressed in each tissue
**Table S27.** Summary of differentially expressed genes (DEGs) for all pairwise comparisons
**Table S28.** Gene identification of the flavonoid and anthocyanin biosynthetic pathways in two *S. macrobotrys* haplotype genomes
**Table S29.** Summary of key enzymes involved in the flavonoid and anthocyanin biosynthetic pathways in *S. macrobotrys*
**Table S30.** Gene expression matrix (log₁₀ CPM) of the anthocyanin and flavonoid pathways in flower and other tissues of *S. macrobotrys*
**Table S31.** Differentially expressed gene (DEG) matrix across floral stages and other tissues in *S. macrobotrys* (1 = significantly downregulated, 2 = significantly upregulated, and 0 = not significant)
**Table S32.** Gene expression matrix (log₁₀ CPM) and significantly differentially expressed genes of the anthocyanin and flavonoid pathways in flower and other tissues of *S. macrobotrys*
**Table S33.** Cumulative expression matrix (CPM values) of all same gene copies encoding enzymes in the anthocyanin and flavonoid pathways across flower and other tissues of two *S. macrobotrys* haplotype genomes: SmA and SmB
**Table S34.** Cumulative expression matrix (log₁₀ CPM) of all same gene copies encoding enzymes in the anthocyanin and flavonoid pathways across flower and other tissues of two *S. macrobotrys* haplotype genomes: SmA and SmB
**Table S35.** List of primers used for qRT‐PCR validation of key genes in *S. macrobotrys*

Materials and Methods
