# Supplementary material for: Haplotype‐resolved telomere‐to‐telomere genome of the jade vine (Strongylodon macrobotrys) provides novel insights into the turquoise flower coloration
Source: J Integr Plant Biol. 2026 Jan 20;68(3):565–7. doi: 10.1111/jipb.70136 (PMC12968347; doi:10.1111/jipb.70136)
Supplement: Supplementary file 1 — Figure S1. Photograph of a chromosome spread from the root tip cells of Strongylodon macrobotrys, with each of the 28 chromosomes clearly labeled for counting and identification Figure S2. Flow cytometry estimation of S. macrobotrys genome size, using Oryza sativa as an internal standard for comparison Figure S3. K‐mer spectrum analyses (k = 19 and k = 21) for the S. macrobotrys genome survey Figure S4. Two‐dimensional heatmaps generated using Smudgeplot (k = 21) illustrate the predicted ploidy of S. macrobotrys Figure S5. Circos plot of the haplotype‐resolved T2T genome assembly of S. macrobotrys Figure S6. Transposable element dynamics in S. macrobotrys Figure S7. Synonymous substitution rate (Ks) distributions reveal whole‐genome duplication and divergence history Figure S8. Boxplot of anthocyanin and saponarin levels across flowering stages (Stage II–IV) based on metabolomic analysis Figure S9. Negative ion mass spectrum of malvin and saponarin Figure S10. UpSet plot summarizing the intersections of expressed genes (CPM > 1) across all tissues77 Figure S11. UpSet plot summarizing the intersections of lowly expressed genes (CPM ≤ 1) across all tissues Figure S12. UpSet plot summarizing the intersections of non‐expressed genes across all tissues Figure S13. Heatmap of gene expression profiles across different tissues Figure S14. Heatmap of gene expression profiles across different tissues (with clustering) Figure S15. Volcano plots showing differentially expressed genes (DEGs) between flower tissues at developmental stages I–IV (FS1–FS4). Figure S16. Summary of downregulated genes (downDEGs) in flower tissues at developmental stages I–IV (abbreviated as FS1–FS4) compared with leaf, root, and stem tissues Figure S17. Summary of upregulated genes (upDEGs) in flower tissues at developmental stages I–IV (FS1–FS4) compared with leaf, root, and stem tissues Figure S18. Significantly enriched KEGG (ko) terms among downDEGs in flower tissues at developmental stages I–IV ( [file JIPB-68-565-s003.docx]

**Supplemental figures:**


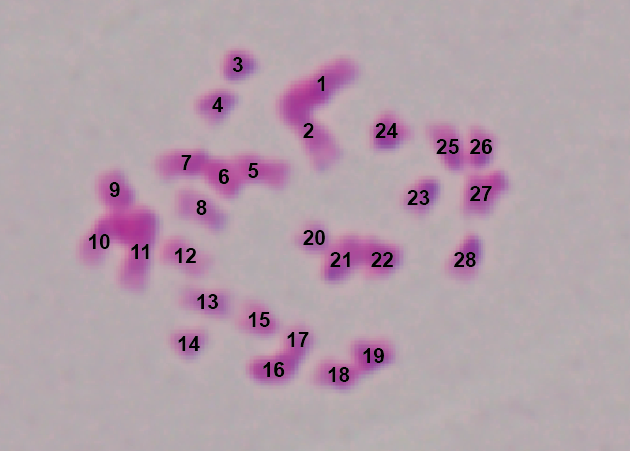


**Figure S1. Photograph of a chromosome spread from the root tip cells of *Strongylodon macrobotrys*, with each of the 28 chromosomes clearly labeled for counting and identification**


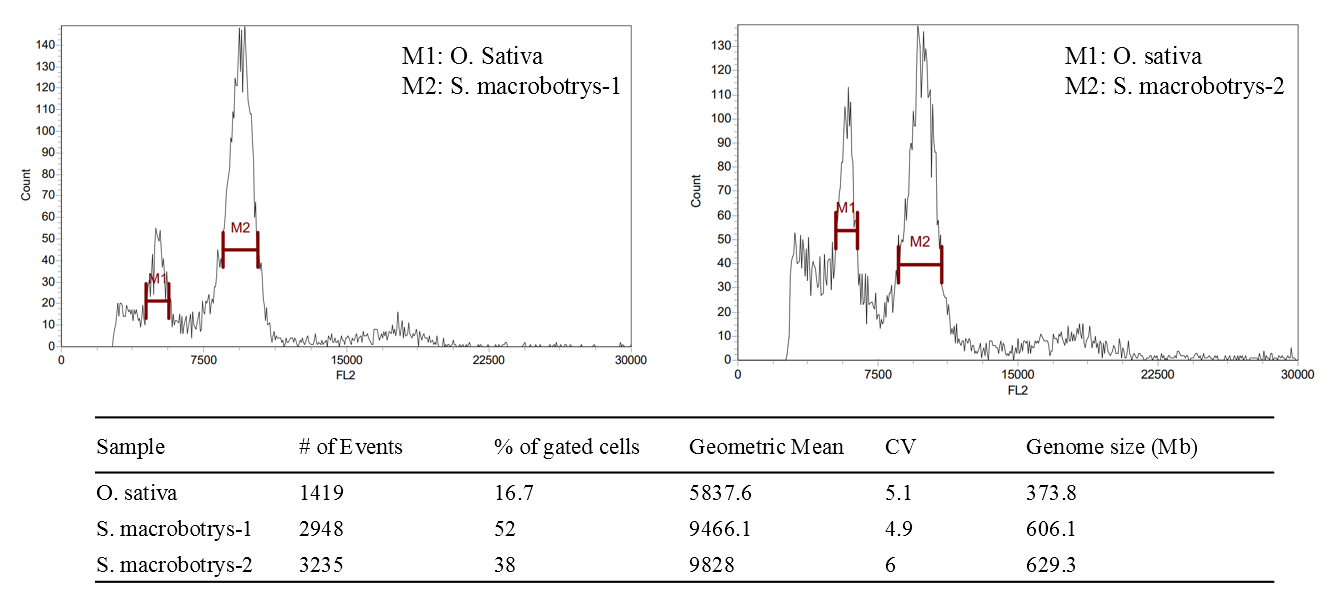


**Figure S2. Flow cytometry estimation of the *S. macrobotrys* genome size, using *Oryza sativa* as an internal standard for comparison**


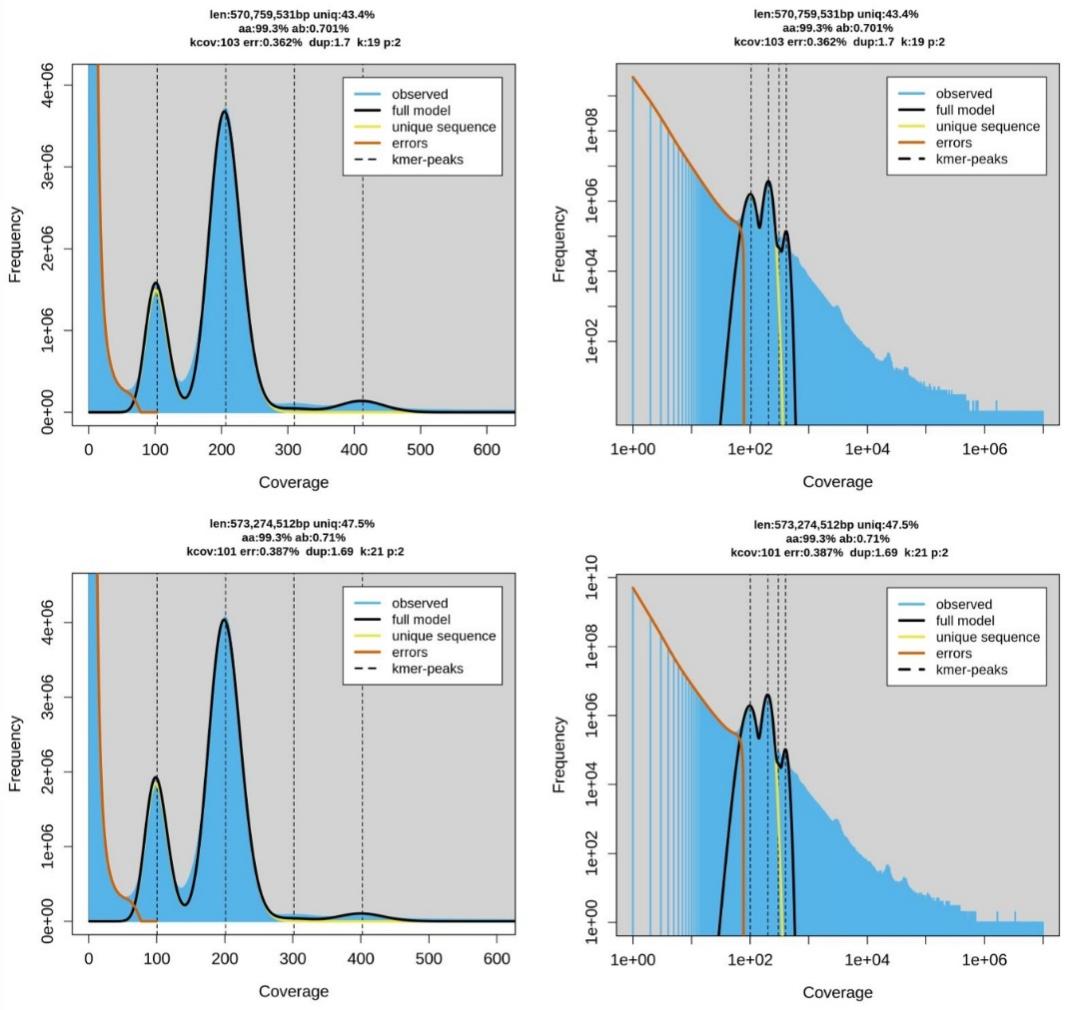


**Figure S3. K-mer spectrum analyses (k = 19 and k = 21) for the *S. macrobotrys* genome survey**

The peaks of both spectra align well with the diploid model (p = 2). The left peak represents heterozygous content (~100× coverage), while the right peak corresponds to homozygous content (~200× coverage). A small peak at ~400× likely indicates repetitive content in the S. macrobotrys genome. Random sequencing errors are observed at coverage near 1.


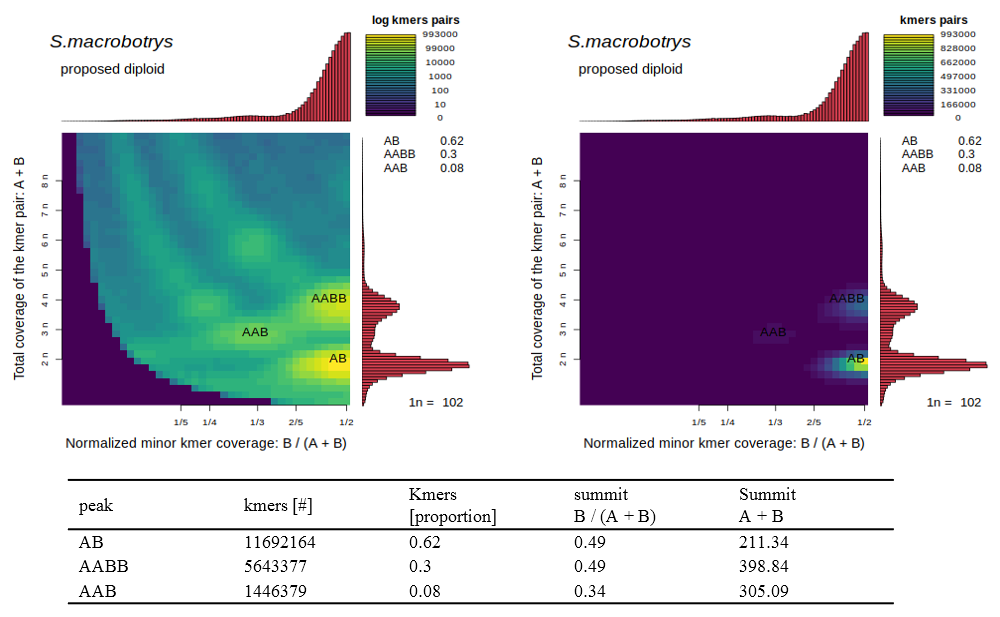


**Figure S4. Two-dimensional heatmaps generated using Smudgeplot (k = 21) illustrate the predicted ploidy of *S. macrobotrys***

Color intensity represents the approximate k-mer count per bin. The estimated ploidy level is indicated in the upper left corner of each graph.


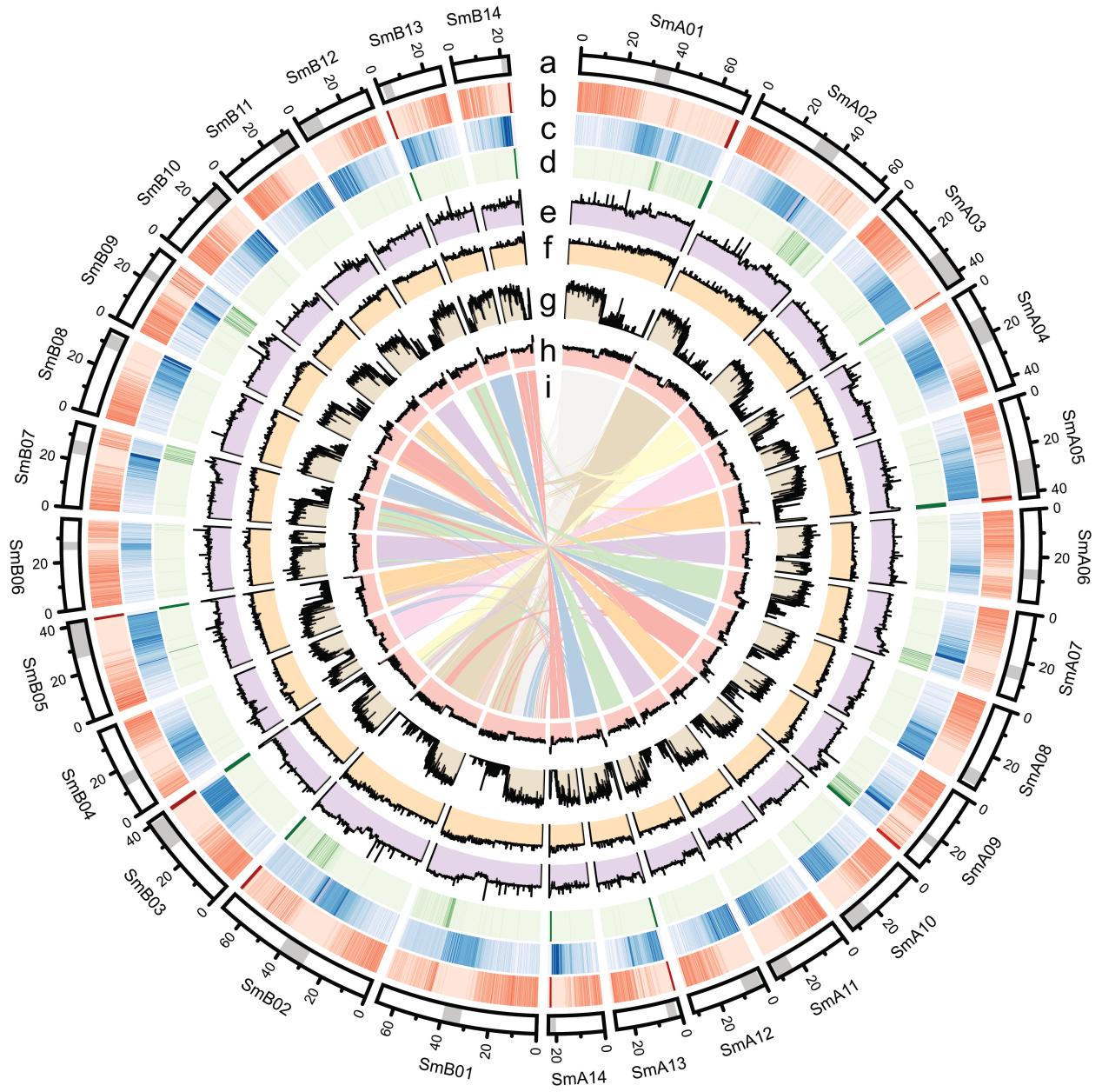


**Figure S5. Circos plot of the haplotype-resolved T2T genome assembly of *S. macrobotrys***

Tracks from outer to inner represent: pseudo-chromosomes with centromeres (grey), gene, TE, non-coding RNA densities, Illumina, HiFi and RNA-seq read coverage (log scale), GC content, and intra-genomic synteny. All statistics are in 100-Kb windows.

**
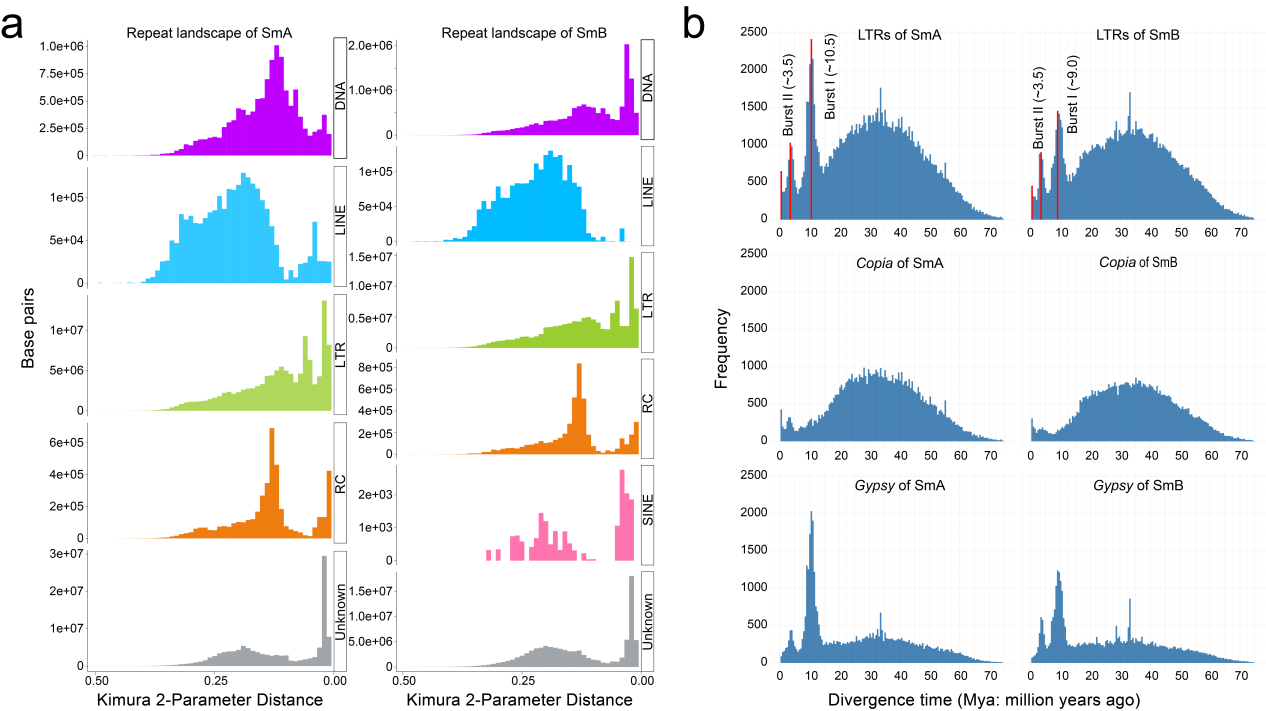
**

**Figure S6. Transposable element dynamics in *S. macrobotrys***

**(a)** K2P distances of TEs from their consensus sequence, reflecting divergence and recent activity. **(b)** Distribution of LTR divergence times from the most recent common ancestor in the haplotype genomes SmA and SmB. Divergence times were estimated by dividing genetic distances by twice the mean substitution rate inferred from the Phaseoleae phylogeny.

**
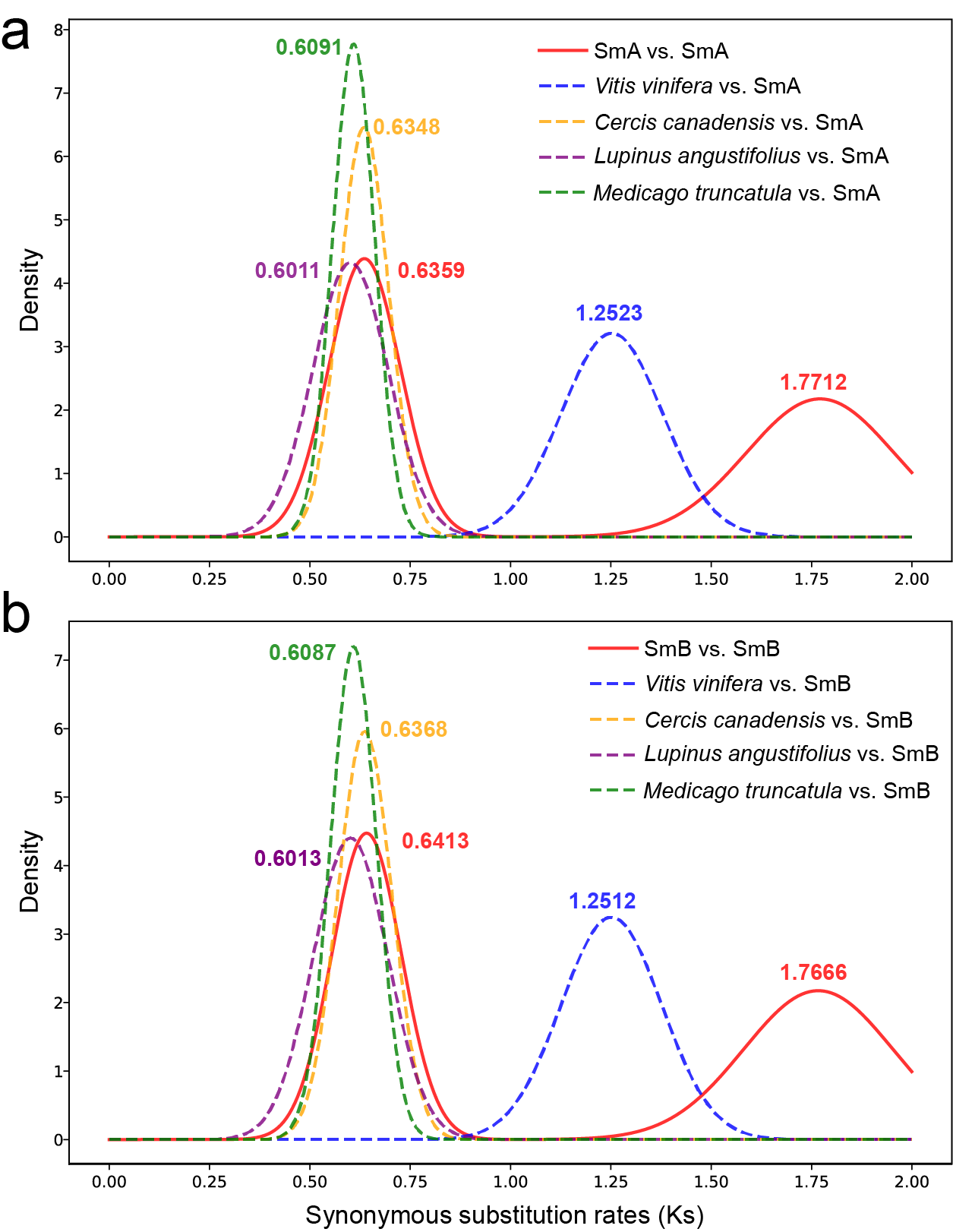
**

**Figure S7. Synonymous substitution rate (Ks) distributions reveal whole-genome duplication and divergence history**

Distributions of synonymous substitution rates (Ks) for paralogous gene pairs within *S. macrobotrys* haplotype genomes SmA (a) and SmB (b), and for orthologous gene pairs between *S. macrobotrys* and related species. For each comparison, the corresponding Ks peak values are labeled near the curves using the same colors as the respective density lines.

**
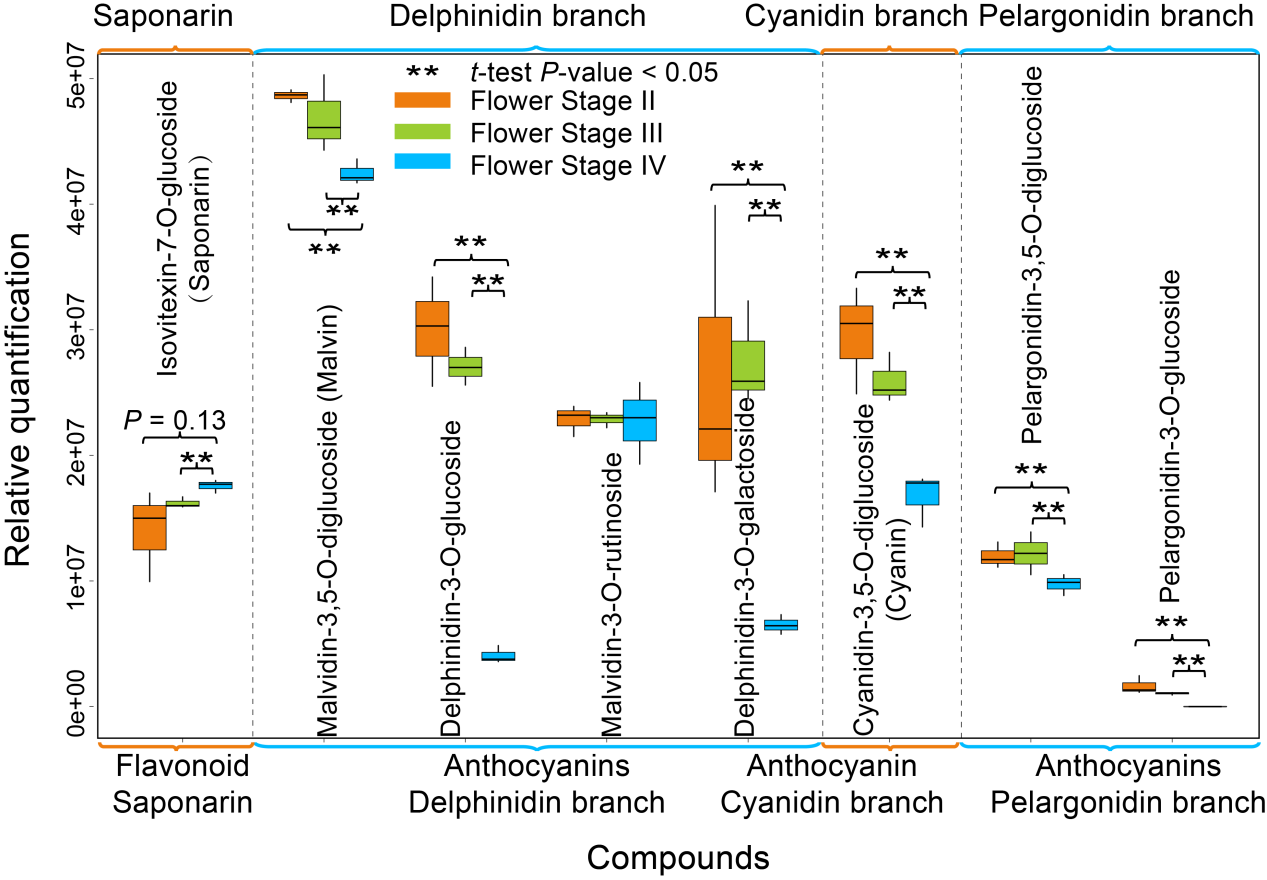
Figure S8. Boxplot of anthocyanin and saponarin levels across flowering stages (Stage II–IV) based on metabolomic analysis**

Differences among stages were assessed using Student’s two-tailed *t*-test, with significance defined as *P-*value < 0.05.


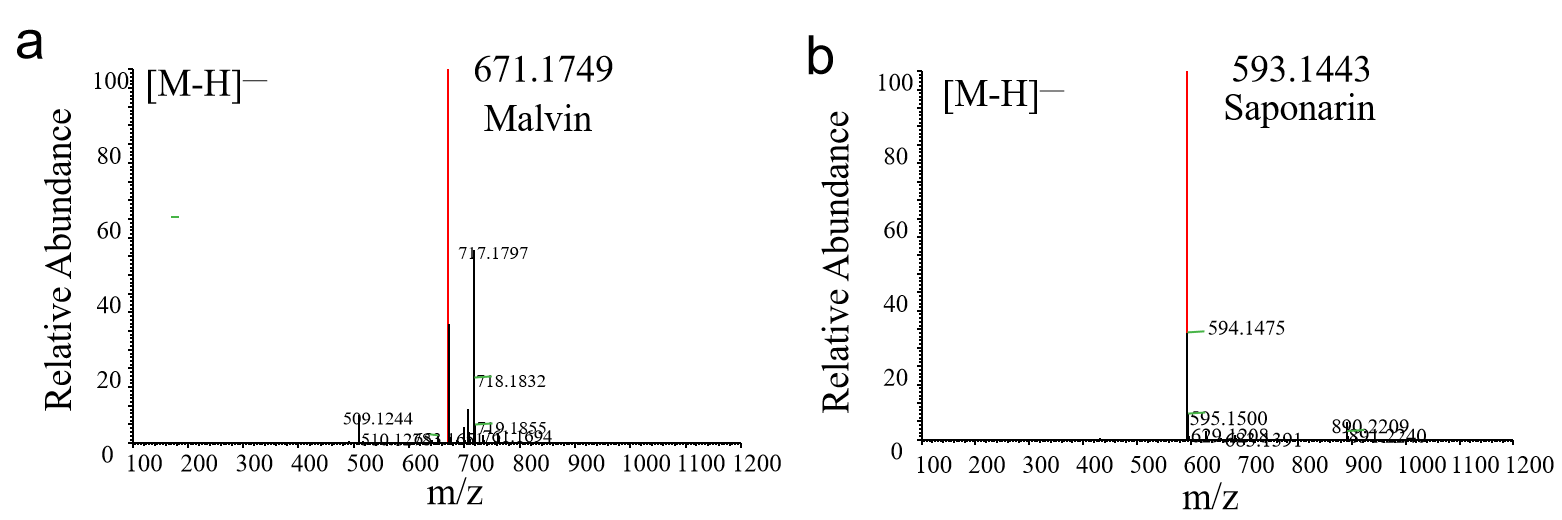


**Figure S9. Negative ion mass spectrum of malvin and saponarin.**

**(a)** Negative ion mode first-order mass spectrum of malvin. The detected deprotonated molecule [M-H]⁻ at m/z 671 corresponds to its theoretical molecular weight. **(b)** Negative ion mode first-order mass spectrum of saponarin. The detected deprotonated molecule [M-H]⁻ at m/z 593 corresponds to its theoretical molecular weight.


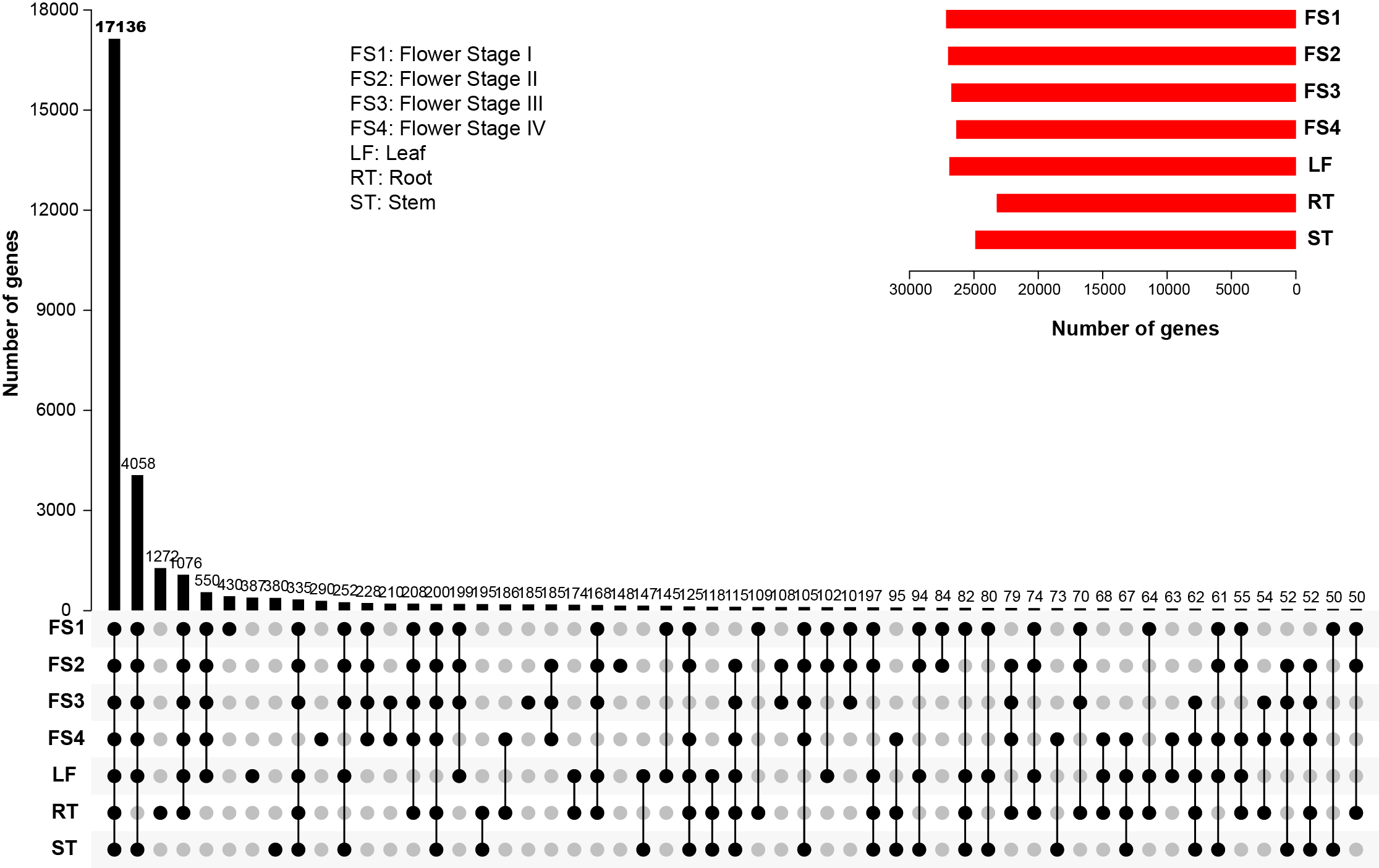


**Figure S10. UpSet plot summarizing the intersections of expressed genes (CPM > 1) across all tissues77**

The bar plot at the top indicates the number of genes in each intersection set, and the dot matrix below shows the corresponding tissues. The horizontal bars in the top-right represent the total number of expressed genes in each individual tissue.


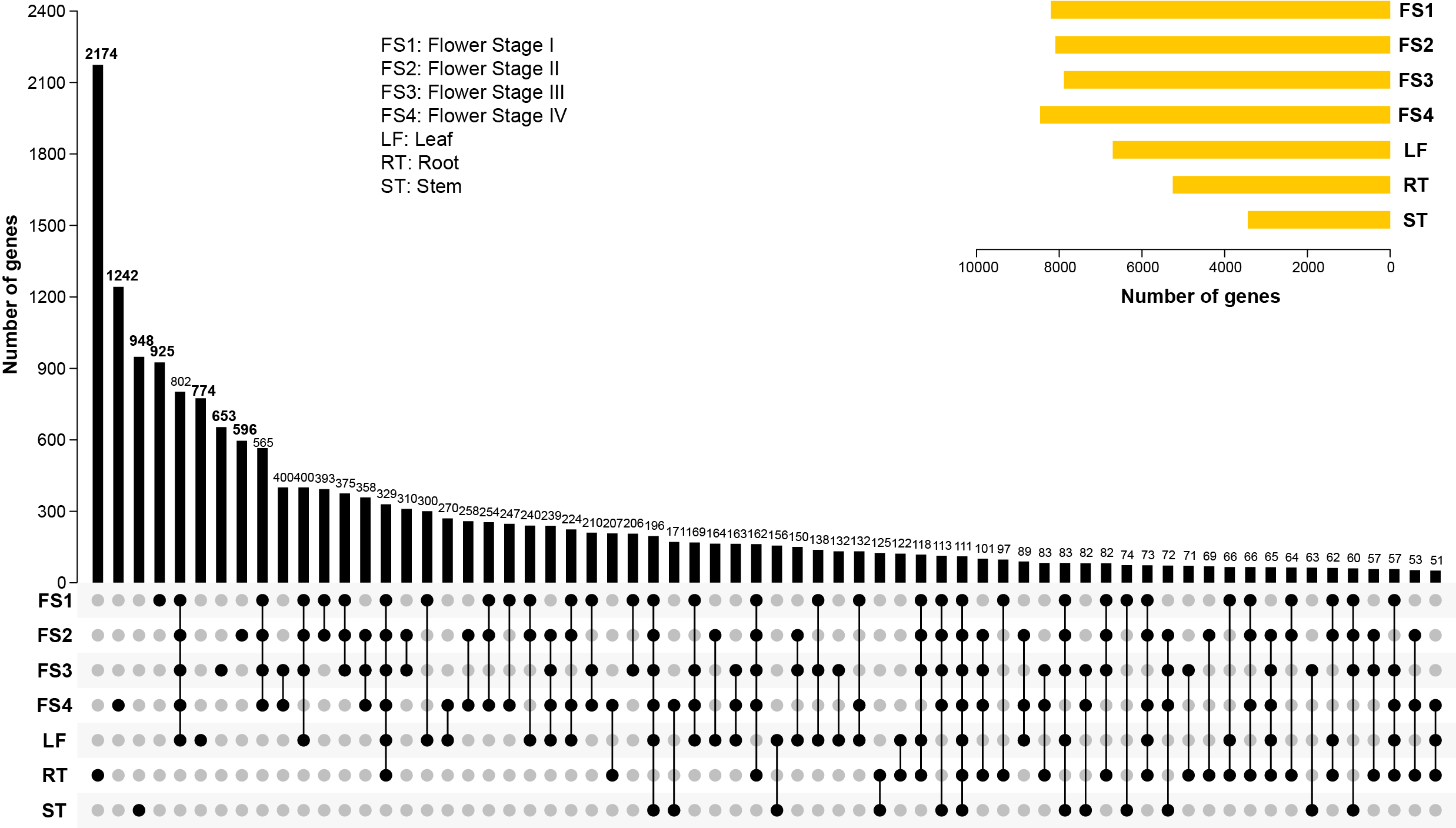


**Figure S11. UpSet plot summarizing the intersections of lowly expressed genes (CPM <= 1) across all tissues**

The bar plot at the top indicates the number of genes in each intersection set, and the dot matrix below shows the corresponding tissues. The horizontal bars in the top-right represent the total number of lowly expressed genes in each individual tissue.


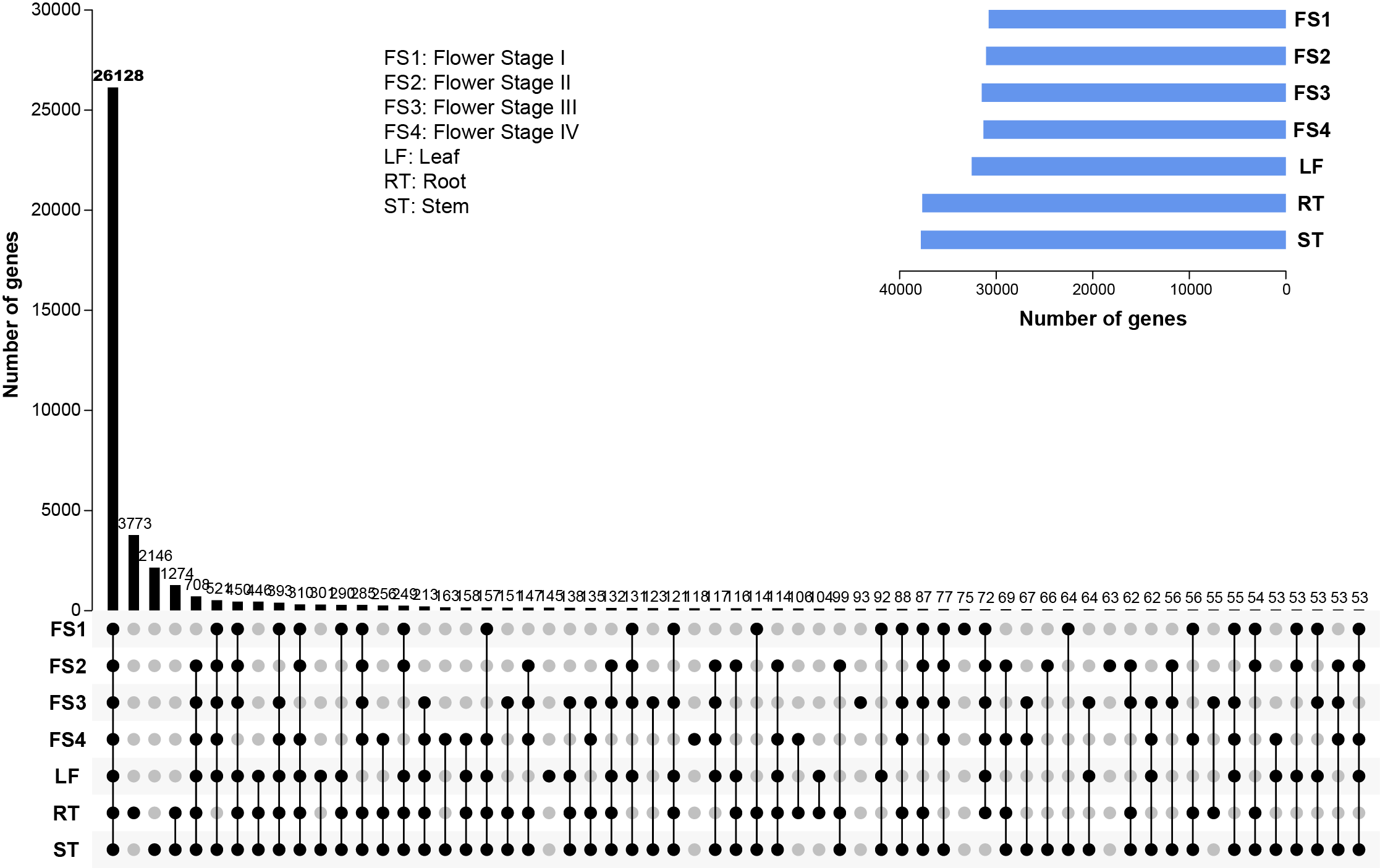


**Figure S12. UpSet plot summarizing the intersections of non-expressed genes across all tissues**

The bar plot at the top indicates the number of genes in each intersection set, and the dot matrix below shows the corresponding tissues. The horizontal bars in the top-right represent the total number of non-expressed genes in each individual tissue.


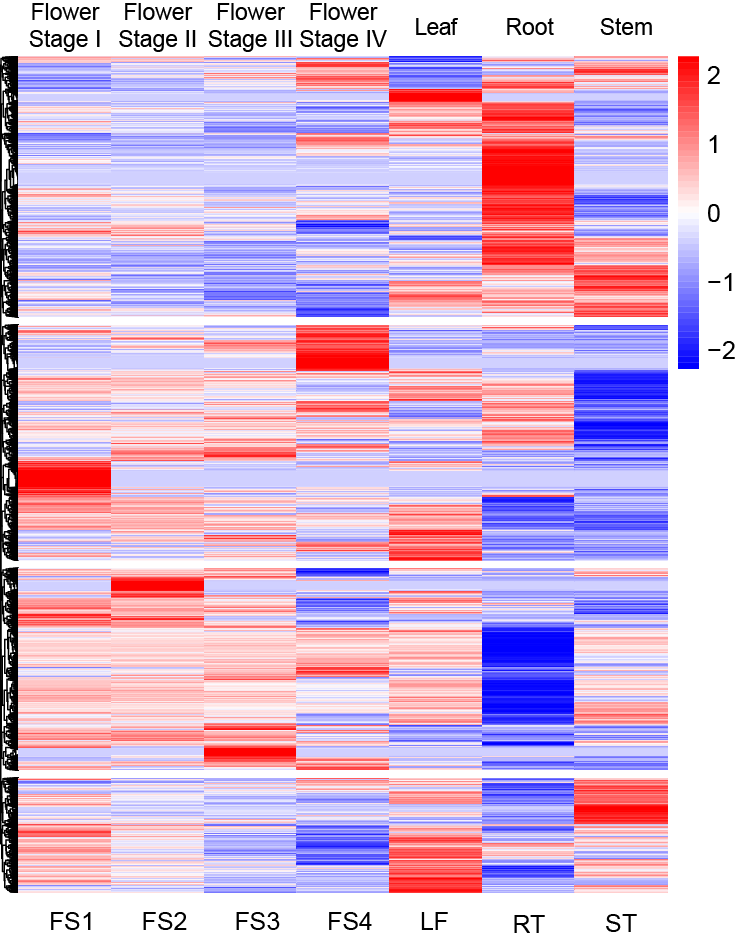


**Figure S13. Heatmap of gene expression profiles across different tissues**

The heatmap displays normalized expression levels of genes across flower stages I to IV, root, leaf, and stem tissues. Gene expression values were normalized by rows (Z-score) to highlight tissue-specific patterns. Genes (rows) were hierarchically clustered to reveal similarities in expression profiles. Red indicates high expression, and blue indicates low expression.

**
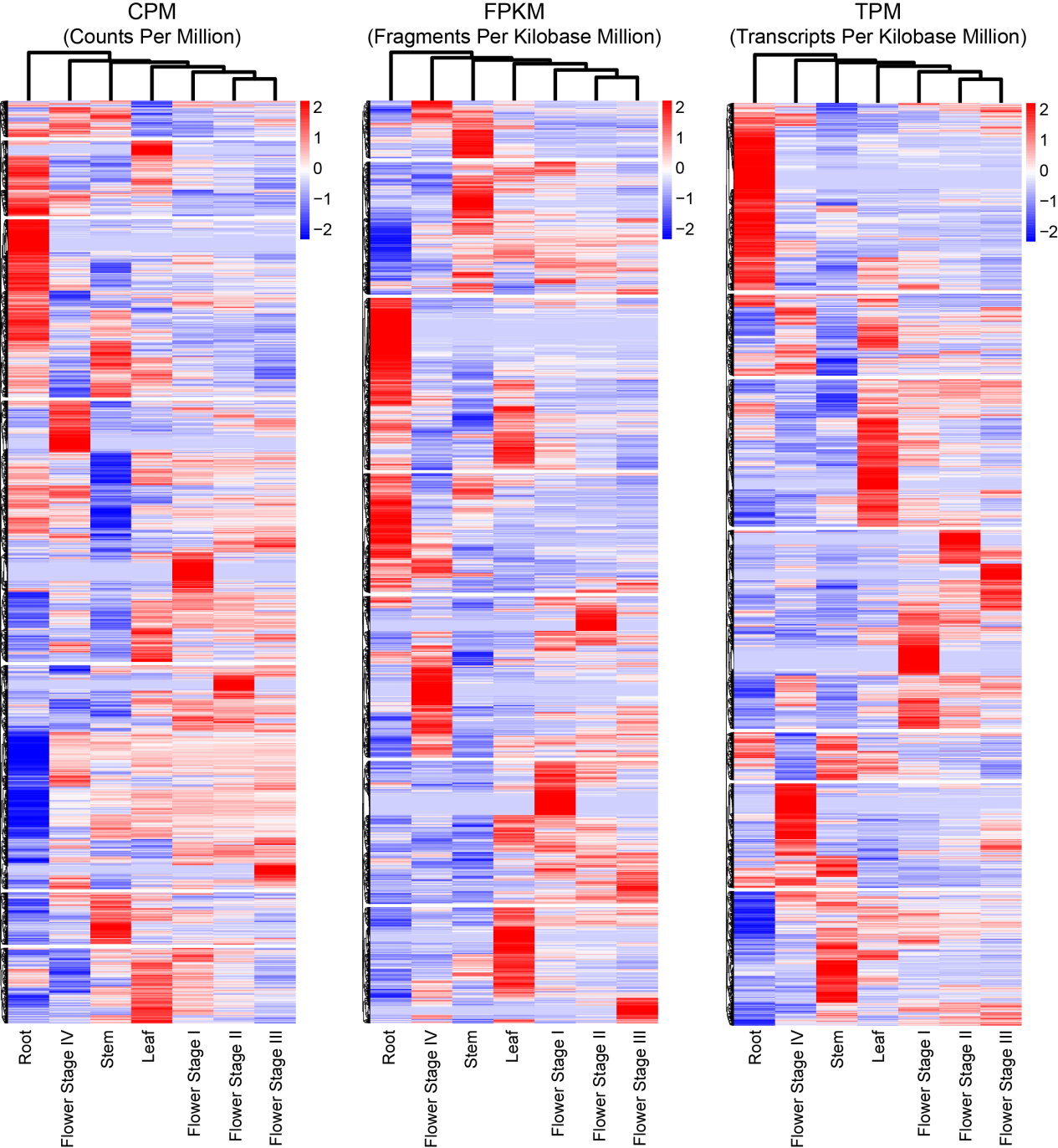
**

**Figure S14. Heatmap of gene expression profiles across different tissues (with clustering)**

The heatmap displays normalized expression levels of genes across flower stages I to IV, root, leaf, and stem tissues. Gene expression values were normalized by rows (Z-score) to highlight tissue-specific patterns. Both genes (rows) and samples (columns) were hierarchically clustered to reveal similarities in expression profiles. Red indicates high expression, and blue indicates low expression.

**
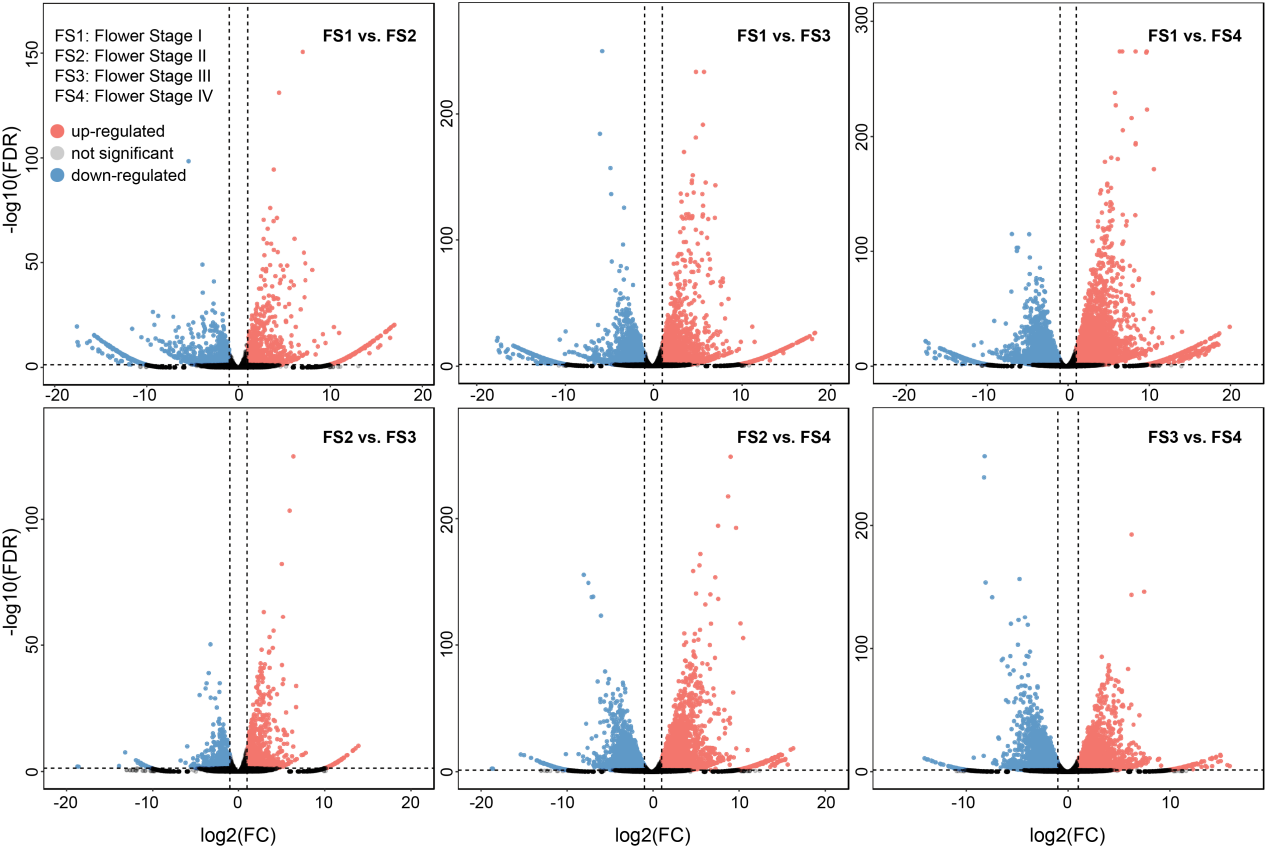
**

**Figure S15. Volcano plots showing differentially expressed genes (DEGs) between flower tissues at developmental stages I–IV (FS1–FS4)**

Each point represents a gene, with red indicating significantly up-regulated genes, blue indicating significantly down-regulated genes, and black indicating non-significant changes. The x-axis represents the log2 fold change (FC), and the y-axis represents the –log10 false discovery rate (FDR). Vertical dashed lines denote the log2(FC) thresholds (|log2FC| > 1), and the horizontal dashed line indicates the significance cutoff (FDR < 0.05).

**
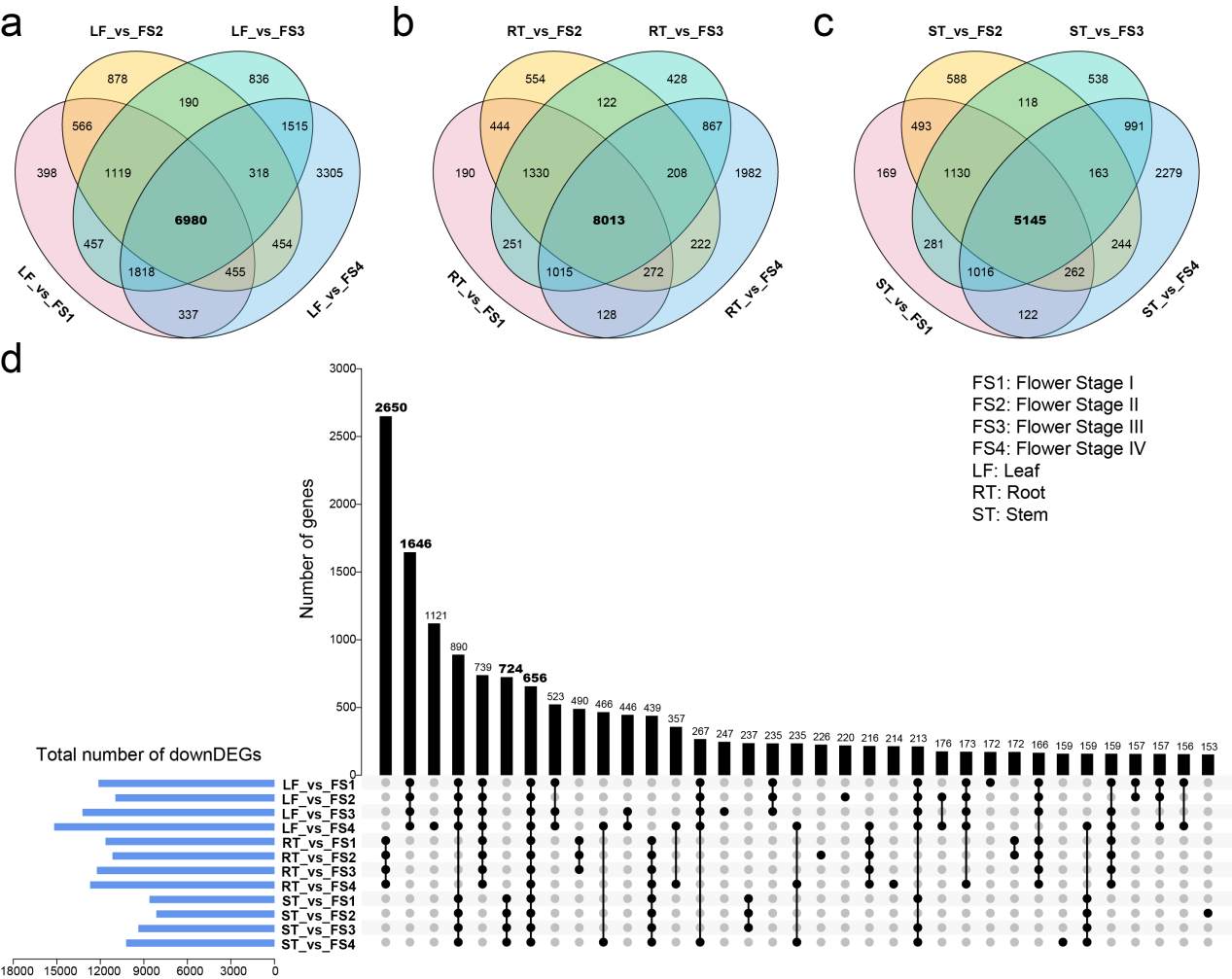
Figure S16. Summary of down-regulated genes (downDEGs) in flower tissues at developmental stages I**–**IV (abbreviated as FS1**–**FS4) compared with leaf, root and stem tissues**

**(a–c)** Venn diagrams showing the overlap of downDEGs in FS1–FS4 when compared with leaf (a), root (b), and stem (c) tissues, respectively. **(d)** UpSet plot summarizing the intersection of downDEGs across all comparisons. The bar plot at the top indicates the number of genes in each intersection set, and the dot matrix below shows the corresponding comparisons. The left horizontal bars represent the total number of downDEGs in each pairwise comparison.

**
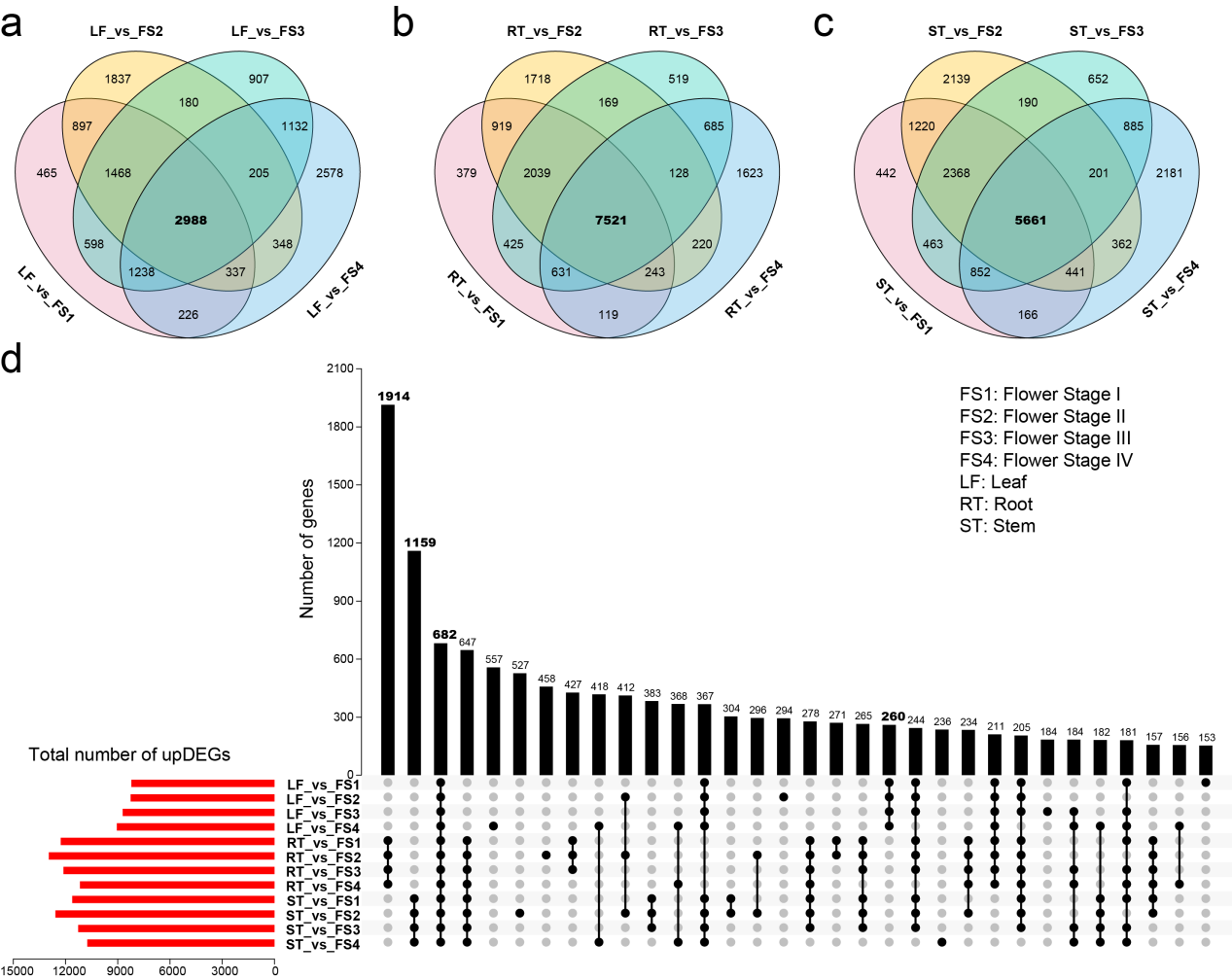
Figure S17. Summary of up-regulated genes (upDEGs) in flower tissues at developmental stages I**–**IV (FS1**–**FS4) compared with leaf, root and stem tissues.** **(a–c)** Venn diagrams showing the overlap of upDEGs in FS1–FS4 when compared with leaf (a), root (b), and stem (c) tissues, respectively. **(d)** UpSet plot summarizing the intersection of upDEGs across all comparisons. The bar plot at the top indicates the number of genes in each intersection set, and the dot matrix below shows the corresponding comparisons. The left horizontal bars represent the total number of upDEGs in each pairwise comparison.


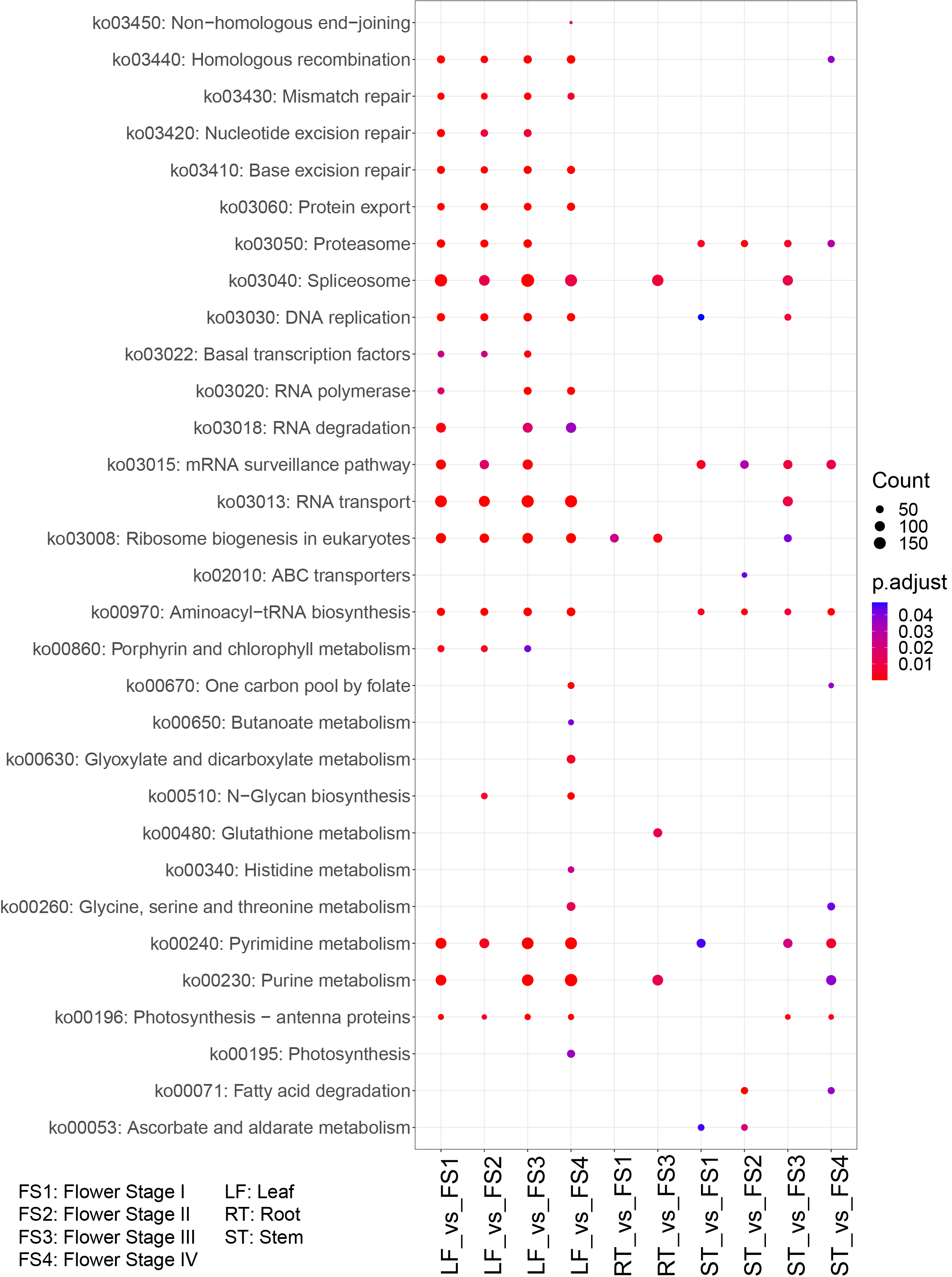


**Figure S18. Significantly enriched KEGG (ko) terms among downDEGs in flower tissues at developmental stages I**–**IV (FS1**–**FS4), identified from pairwise comparisons indicated along the X-axis**

Enrichment analysis was performed using the R package clusterProfiler, and only terms with p.adjust < 0.05 and qvalue < 0.05 were considered significant.


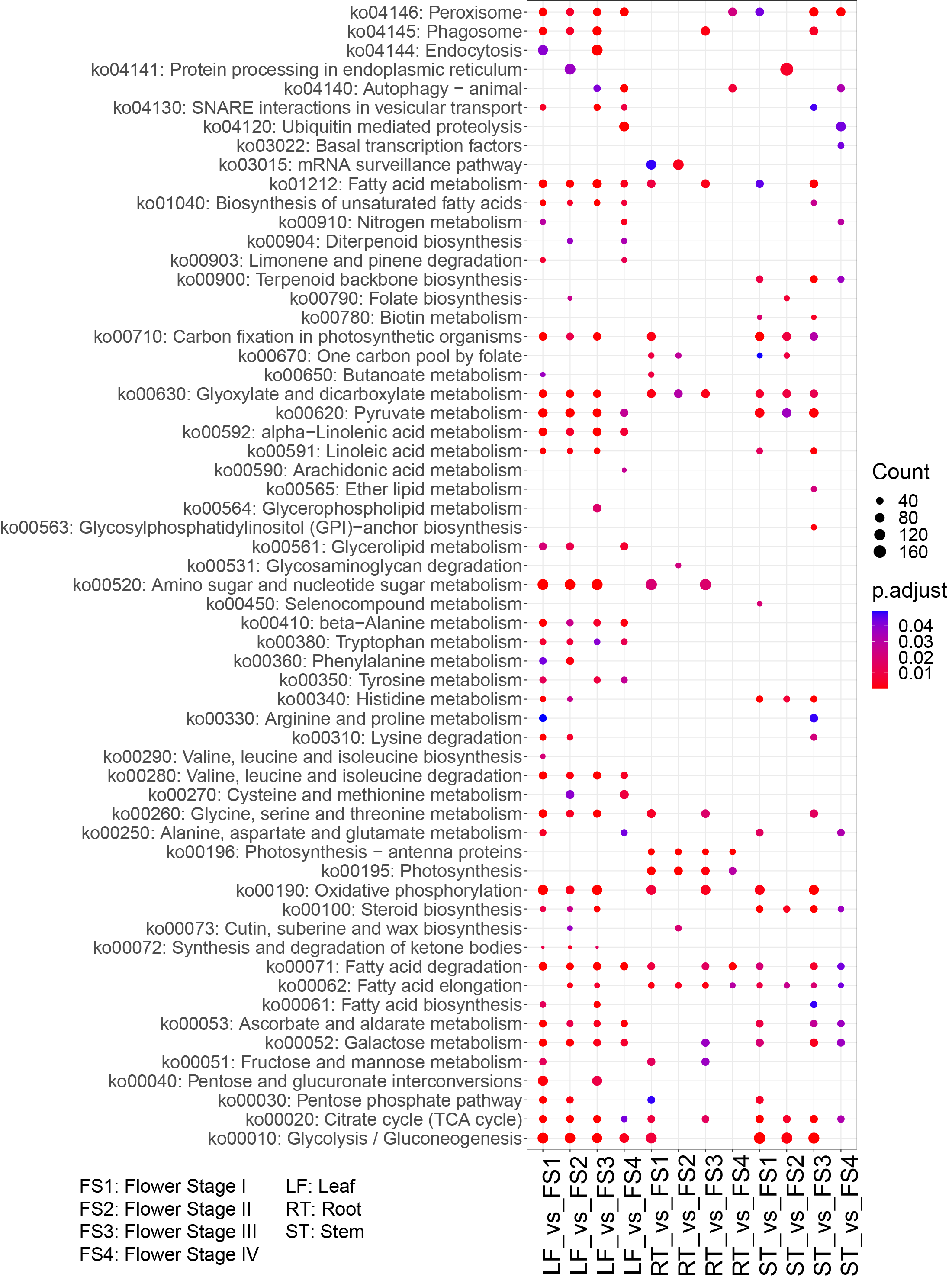


**Figure S19. Significantly enriched KEGG (ko) terms among upDEGs in flower tissues at developmental stages I**–**IV (FS1**–**FS4), identified from pairwise comparisons indicated along the X-axis.**

Enrichment analysis was performed using the R package clusterProfiler, and only terms with p.adjust < 0.05 and qvalue < 0.05 were considered significant.

**
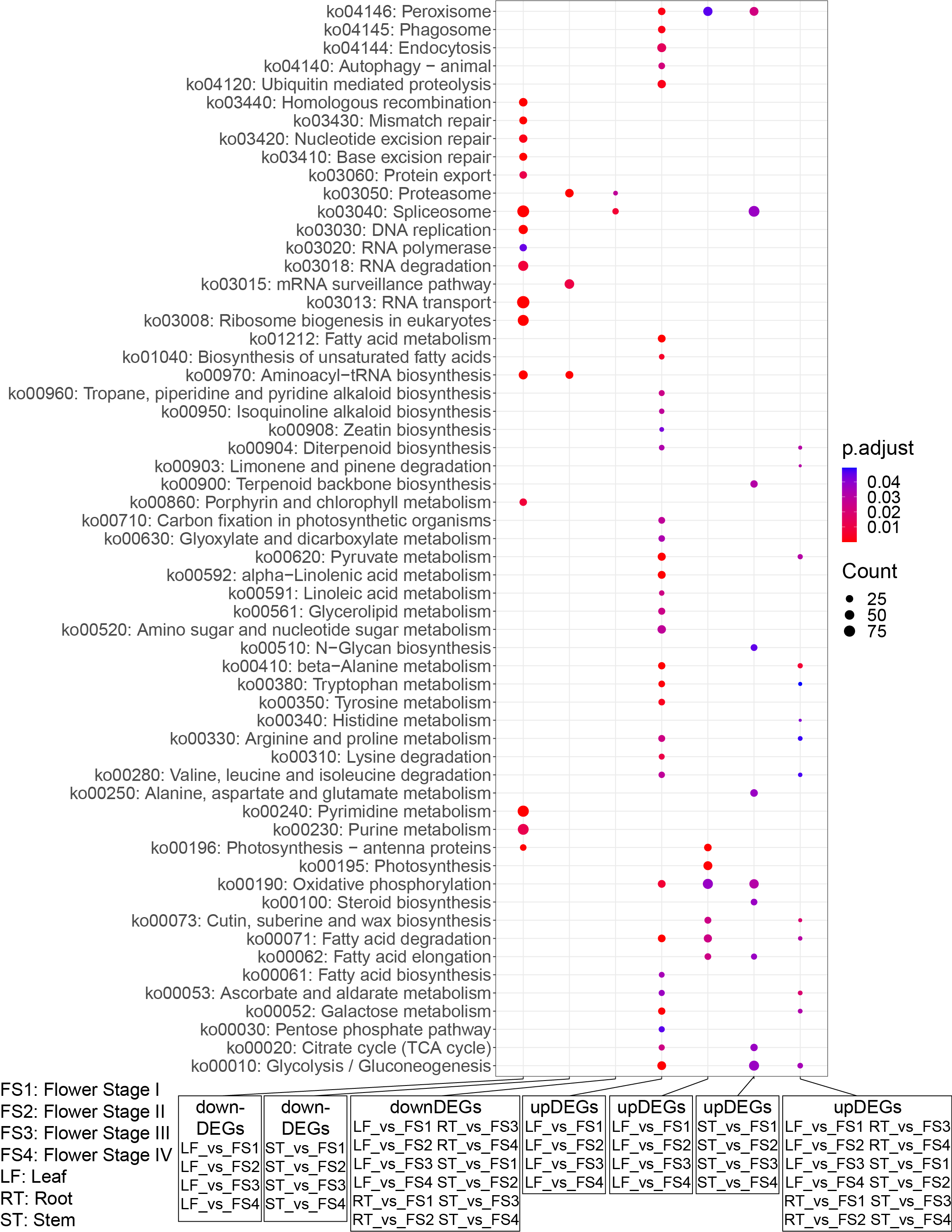
**

**Figure S20. Significantly enriched KEGG (ko) terms among shared downDEGs and upDEGs in flower tissues at developmental stages I**–**IV (FS1**–**FS4), identified from pairwise comparisons indicated along the X-axis.**

Enrichment analysis was performed using the R package clusterProfiler, and only terms with p.adjust < 0.05 and qvalue < 0.05 were considered significant.


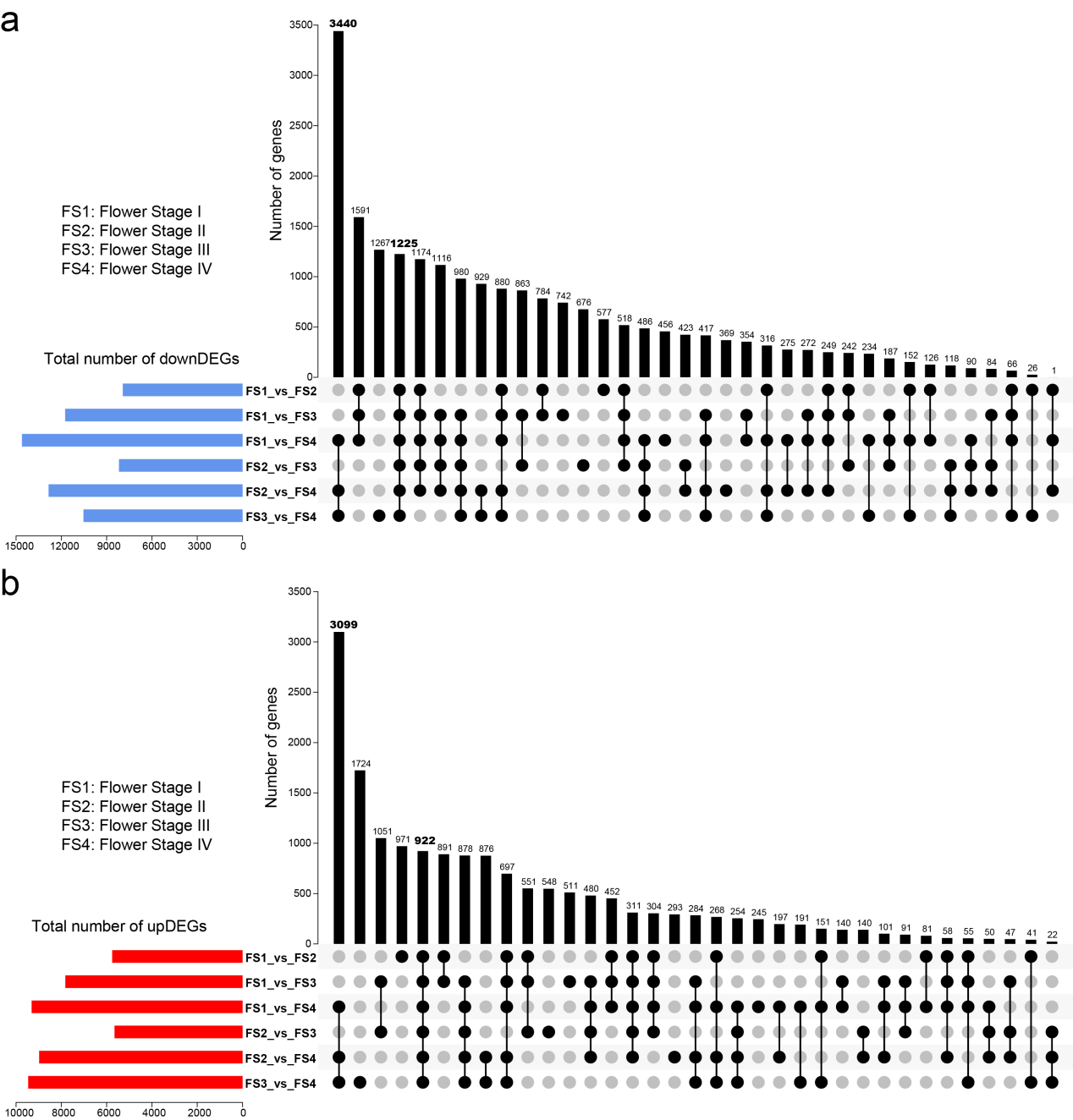


**Figure S21. UpSet plot summarizing the intersection of downDEGs (a) and upDEGs (b) across all pairwise comparisons among flower tissues at different developmental stages (FS1**–**FS4).**

The bar plot at the top indicates the number of genes in each intersection set, and the dot matrix below shows the corresponding comparisons. The horizontal bars on the left represent the total number of up- or down-regulated genes in each pairwise comparison.

**
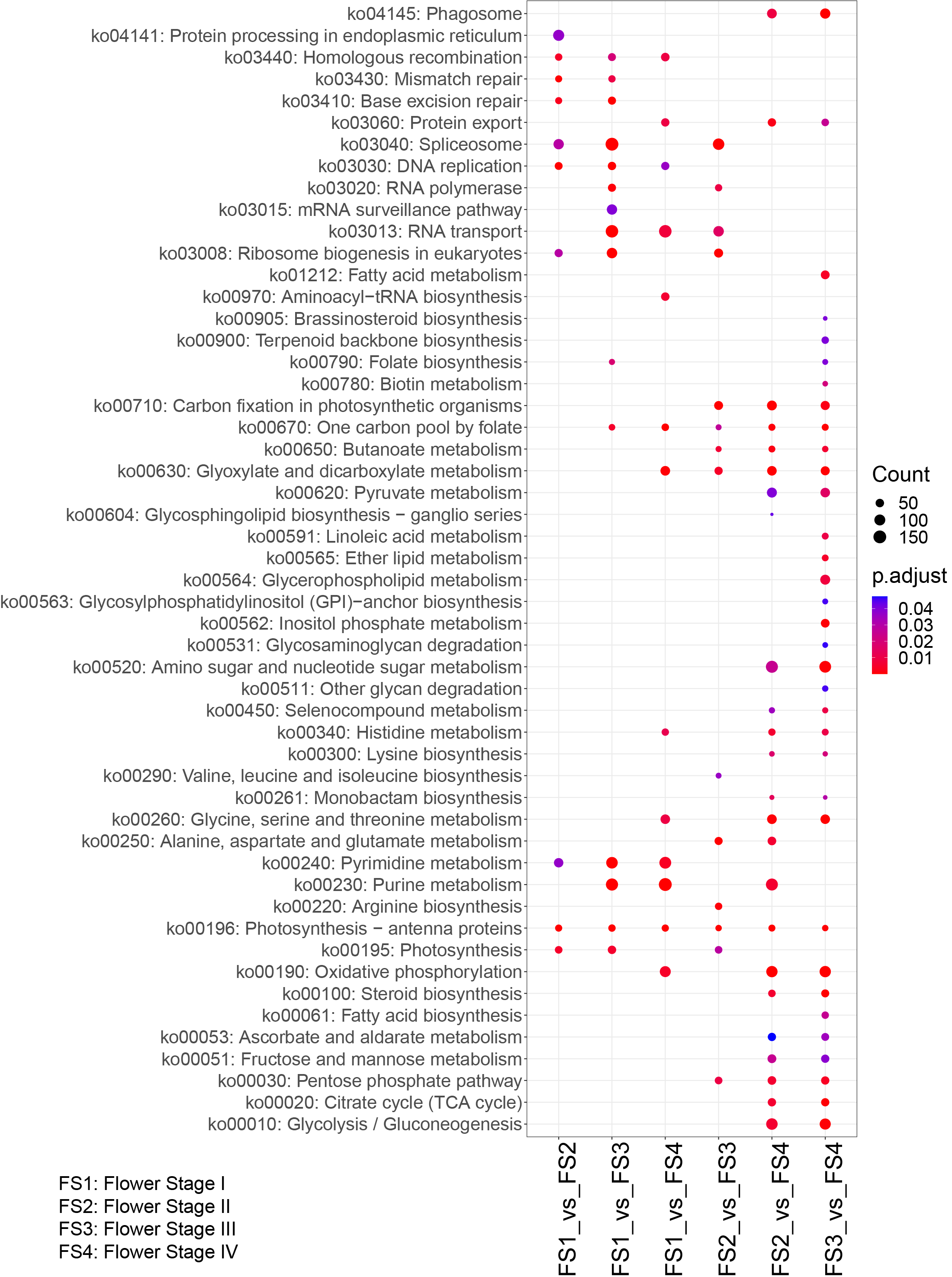
Figure S22. Significantly enriched KEGG (ko) terms among upDEGs during flower development, identified from pairwise comparisons indicated along the X-axis.**

Enrichment analysis was performed using the R package clusterProfiler, and only terms with p.adjust < 0.05 and qvalue < 0.05 were considered significant.

**
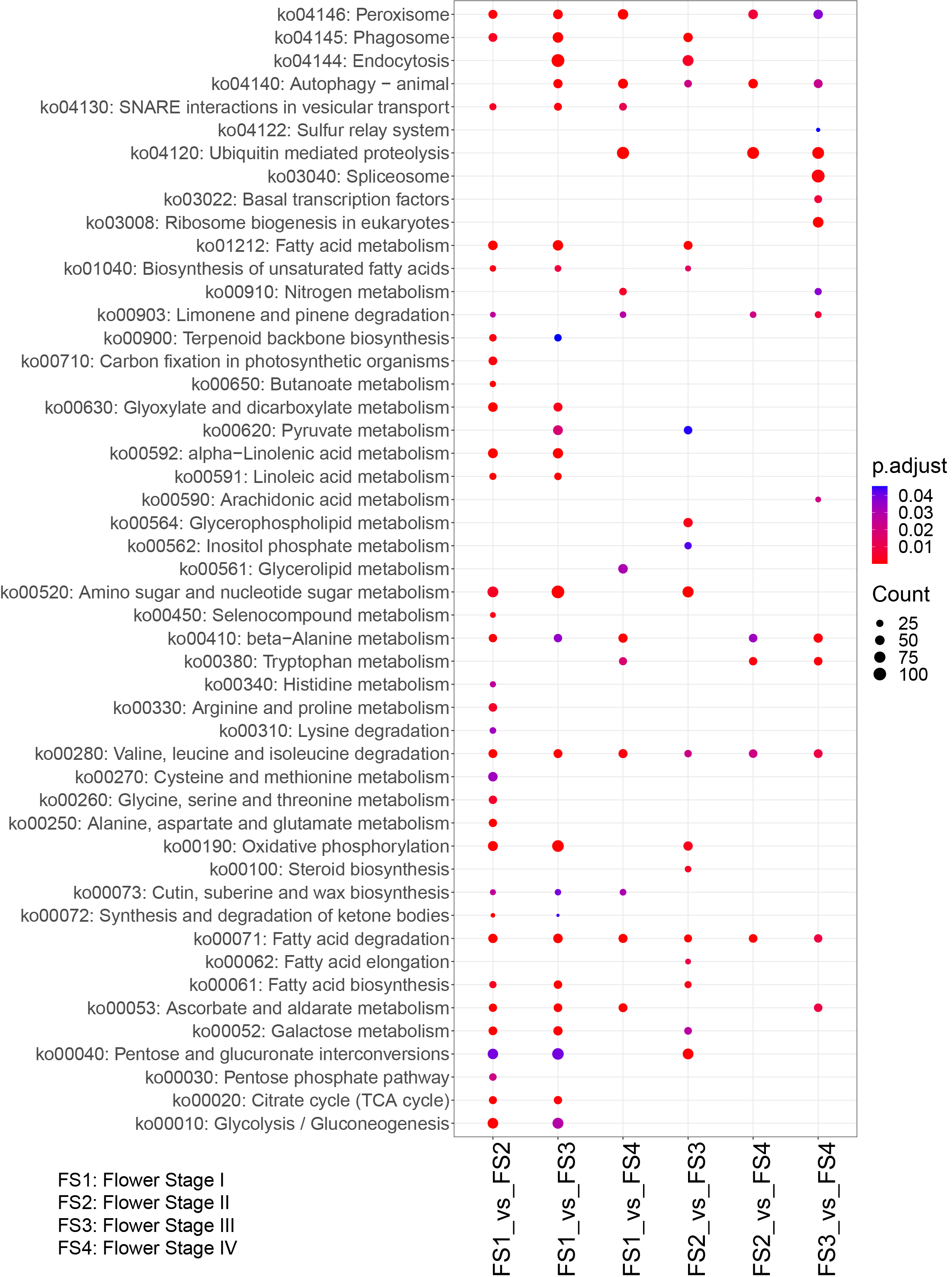
**

**Figure S23. Significantly enriched KEGG (ko) terms among upDEGs during flower development, identified from pairwise comparisons indicated along the X-axis.**

Enrichment analysis was performed using the R package clusterProfiler, and only terms with p.adjust < 0.05 and qvalue < 0.05 were considered significant.

**
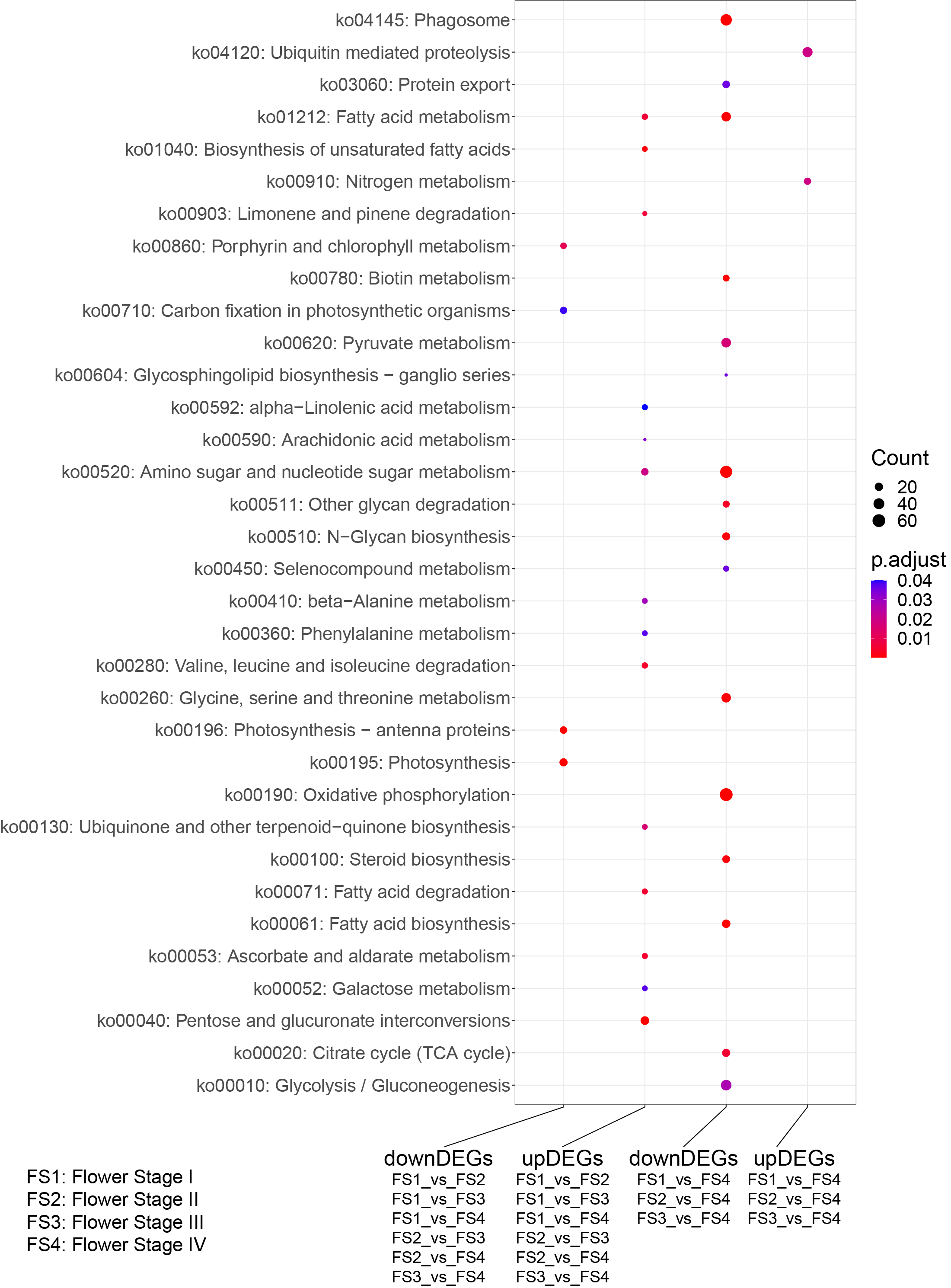
**

**Figure S24. Significantly enriched KEGG (ko) terms among shared downDEGs and upDEGs during flower development, identified from pairwise comparisons indicated along the X-axis.**

Enrichment analysis was performed using the R package clusterProfiler, and only terms with p.adjust < 0.05 and qvalue < 0.05 were considered significant.

**
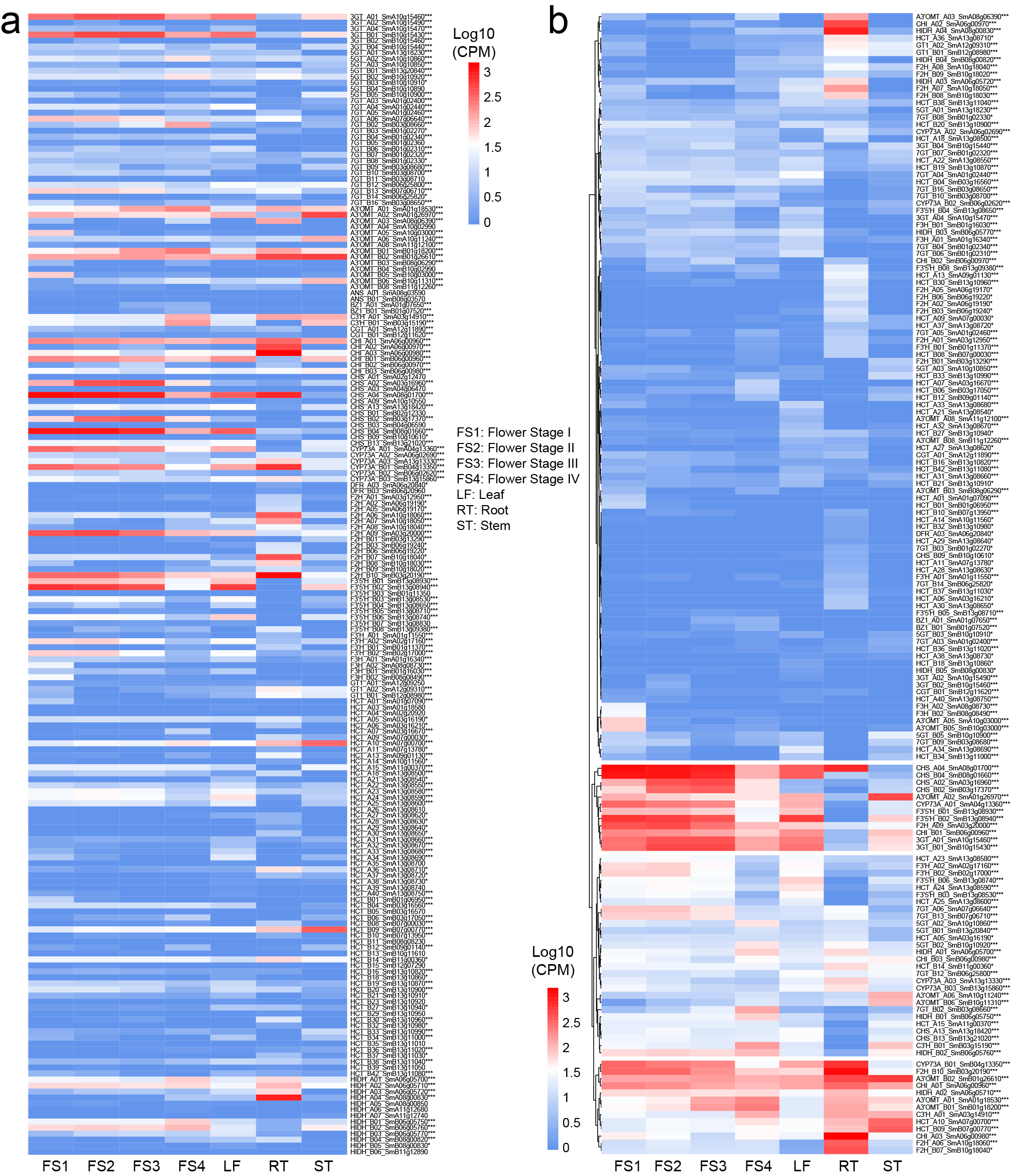
**

**Figure S25. Heatmaps showing gene expression profiles across various tissues.**

**(a)** Heatmap of the expression levels of 241 genes associated with anthocyanin and flavone biosynthetic pathways. **(b)** Heatmap of the expression levels of 241 differentially expressed genes (DEGs) within the same pathways. Expression levels were normalized as Log10(CPM) to enhance visualization. Red indicates high expression, while blue indicates low expression. The heatmaps encompass gene expression across flower developmental stages I to IV (FS1–FS4), root, leaf, and stem tissues. In panel (b), genes (rows) were hierarchically clustered to reveal expression pattern similarities. Asterisks following gene names indicate significance of differential expression: * —significantly differentially expressed only between flowers and other tissues; ** —significantly differentially expressed only among floral developmental stages; *** —significantly differentially expressed both among floral developmental stages.
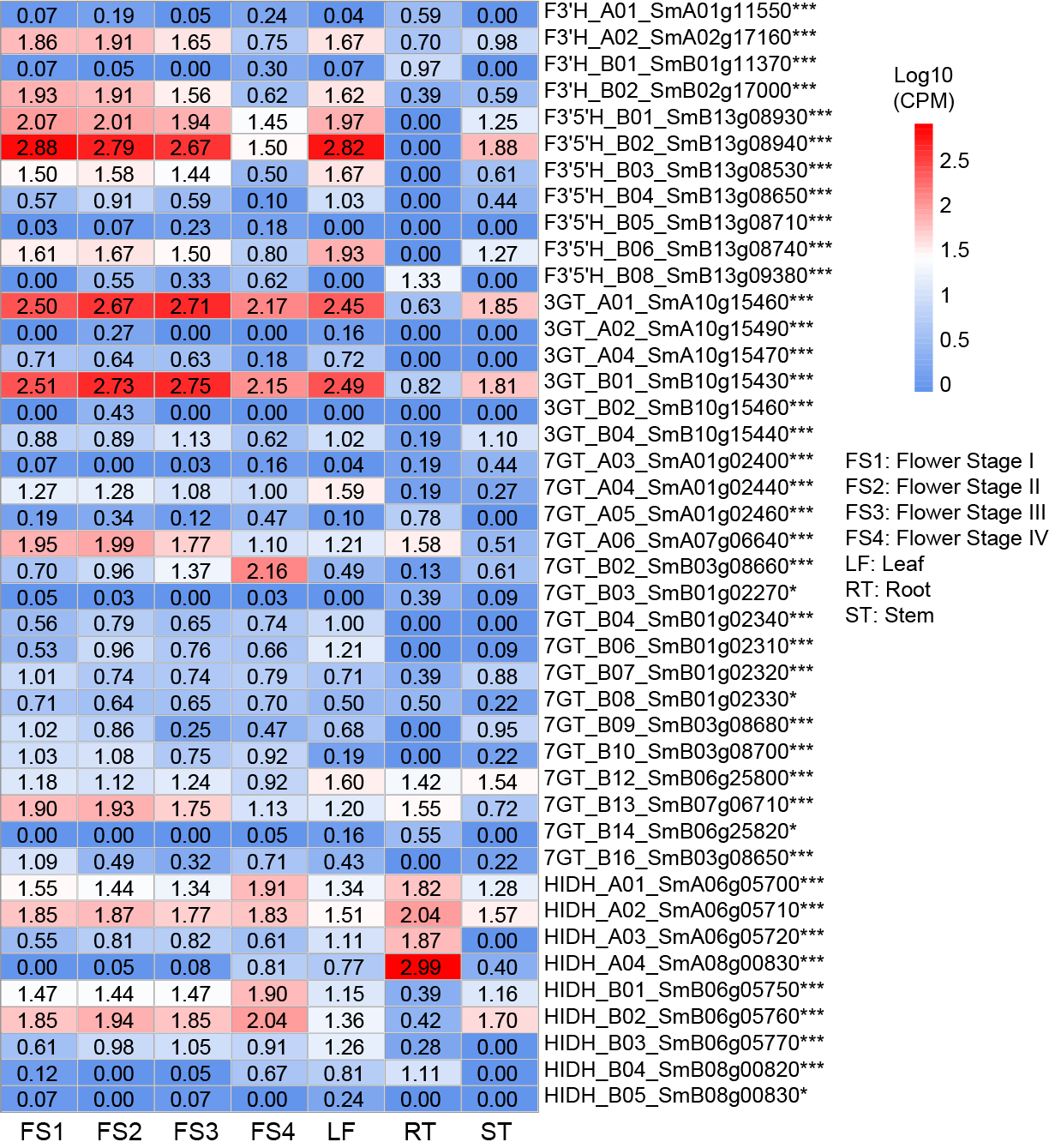


**Figure S26. Heatmap of the expression levels of five enzyme genes involved in the anthocyanin and flavone biosynthetic pathways.**

Expression levels were normalized as Log10(CPM) to enhance visualization, with corresponding Log10(CPM) values labeled within the heatmap. Red indicates high expression, while blue indicates low expression. The heatmaps encompass gene expression across flower developmental stages I to IV (FS1–FS4), root, leaf, and stem tissues. Asterisks following gene names indicate significance of differential expression: * —significantly differentially expressed only between flowers and other tissues; ** —significantly differentially expressed only among floral developmental stages; *** —significantly differentially expressed both among floral developmental stages. and between flowers and other tissues.


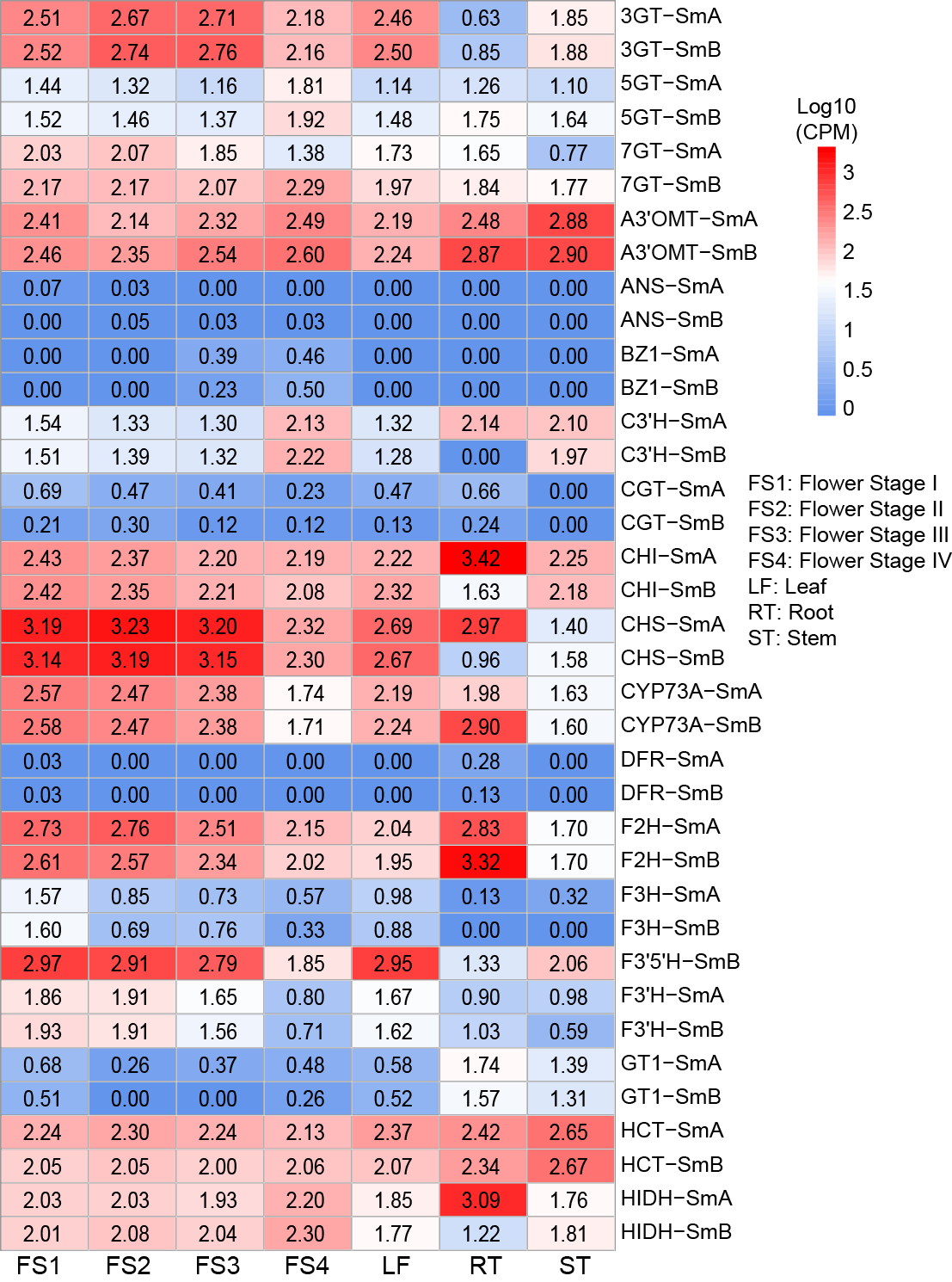


**Figure S27. Heatmap of the cumulative expression levels of all same gene copies encoding enzymes associated with anthocyanin and flavonoid biosynthetic pathways of two *S. macrobotrys* haplotype genomes SmA and SmB.**

Expression levels were normalized as Log10(CPM) to enhance visualization, with corresponding Log10(CPM) values labeled within the heatmap. Red indicates high expression, while blue indicates low expression. The heatmap encompasses gene expression across flower developmental stages I to IV (FS1–FS4), root, leaf, and stem tissues.

**
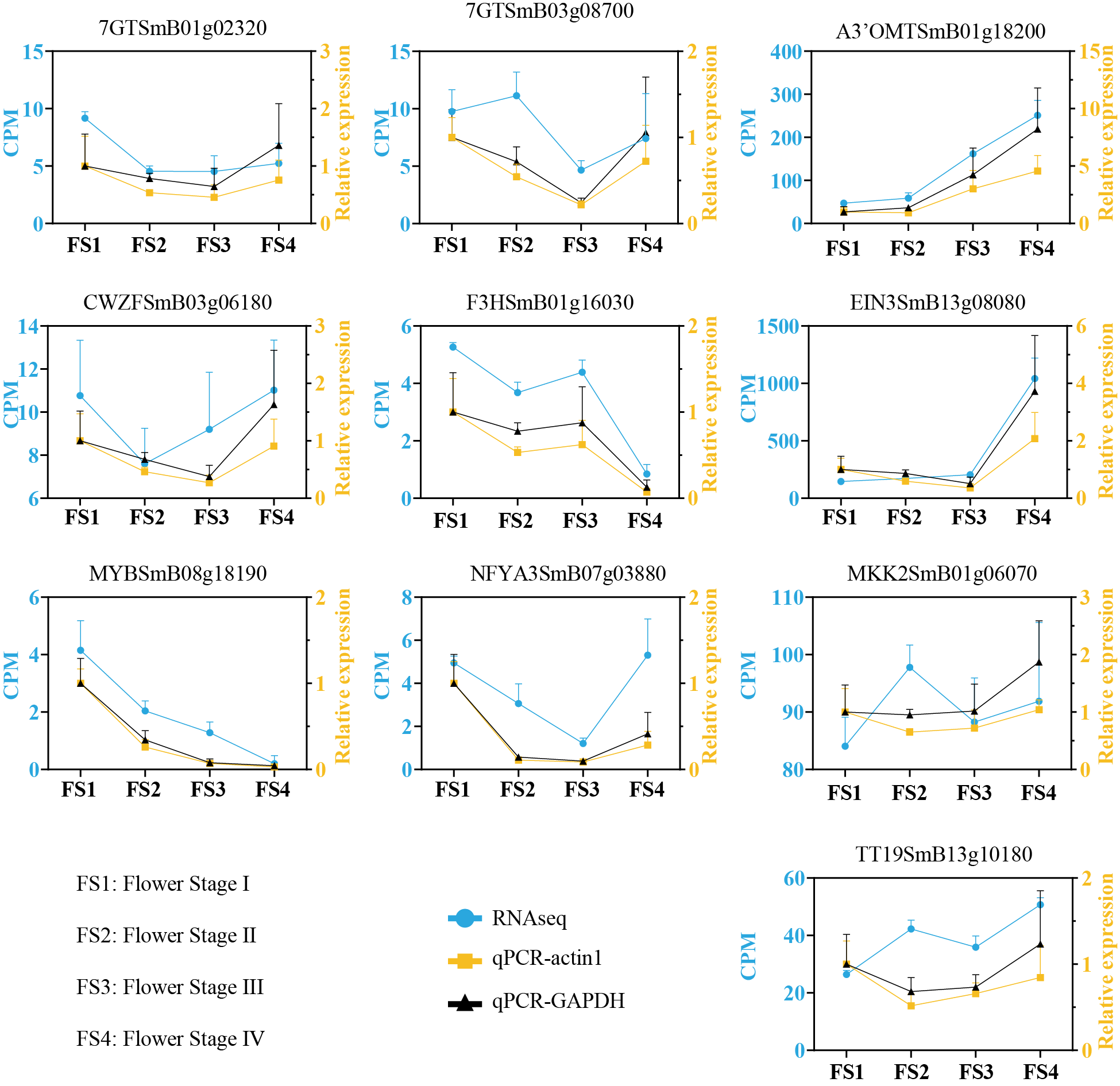
**

**Figure S28. Quantitative real-time polymerase chain reaction (qRT-PCR) analysis performed to confirm the transcriptomic data.**

To validate the gene expression, nine genes were selected for qRT-PCR analysis using *Smactin1* and *SmGAPDH* as reference genes in this study.
