# Supplementary material for: Haplotype‐resolved telomere‐to‐telomere genome of the jade vine (Strongylodon macrobotrys) provides novel insights into the turquoise flower coloration
Source: J Integr Plant Biol. 2026 Jan 20;68(3):565–7. doi: 10.1111/jipb.70136 (PMC12968347; doi:10.1111/jipb.70136)
Supplement: Supplementary file 3 — Materials and Methods [file JIPB-68-565-s001.docx]

**Data availability**

The raw sequencing data generated in this study has been deposited to the GSA database under the accession number CRA025976 (https://ngdc.cncb.ac.cn/). The genome and annotations are accessible via Figshare (https://doi.org/10.6084/m9.figshare.29105318). In addition, the assembled genomes have been deposited in NCBI under accession numbers JBQXYP000000000 and JBQXYQ000000000.

Materials and Methods

Plant Material Resources

The *Strongylodon macrobotrys* plants used in this study were grown in the greenhouse of the South China Botanical Garden, Chinese Academy of Sciences. In late March, young leaves were collected and cleaned with absolute ethanol for whole genome sequencing and assembly. Flowers, roots and stems were also collected for genome annotation, gene expression analysis, and functional studies. To investigate the development of floral organs in pendant racemes or pseudoracemes, flowers at four different stages were sampled from the bottom to the top of each inflorescence. All samples were immediately frozen in liquid nitrogen and stored on dry ice. To ensure reproducibility in functional analysis, three biological replicates were applied, each analysis from a separate individual plant following the same procedure. Voucher specimens (Sm_SCBG) have been deposited in the herbarium of the South China Botanical Garden.

Karyotype Analysis

To analyze the karyotype of *S. macrobotrys*, the chromosome number was determined using the squash technique on root apical meristem cells from seedlings. Seeds were germinated until roots reached 10-12 mm in length. Root tips were pre-treated with an aqueous bromonaphthalene solution for 3.5 hours at 40℃, then fixed in a solution of 95% ethanol and 45% acetic acid. The samples were stained with aceto-haematoxylin, and slides were prepared using the squash method in 80% chloral hydrate. Images were captured using a microscope with immersion oil at 100×16 magnification. Chromosomes were counted in five mitotic cells per slide.

Flow Cytometry

Flow cytometry was performed following the protocol of (Dolezel and Bartos, 2005), using *Glycine max* (Williams 82; 1,115 Mb) as the internal reference standard. Fresh leaf tissue was finely chopped and incubated in a suspension buffer for 5 minutes, then stained with propidium iodide for 30 minutes. RNase A was added to digest RNA. The resulting nuclei suspensions were analyzed using a Sysmex CyFlow Cube 6 flow cytometer. Genome size was estimated by comparing the fluorescence intensity of *S. macrobotrys* to that of the reference standard.

Whole genome and Hi-C sequencing

A preliminary genome survey of *S. macrobotrys* was conducted using whole-genome shotgun (WGS) sequencing. Genomic DNA was extracted from leaf tissue using a modified CTAB method. Sequencing was performed on the Illumina NovaSeq 6000 platform, producing approximately 235× coverage from a 2×150 bp paired-end library (**Table S1**). Raw reads were filtered by fastp v0.23.3 (Chen et al., 2018) to remove adapters and low-quality sequences. A k-mer frequency distribution was generated using Jellyfish v2.3.0 (Marcais and Kingsford, 2011), and genome features were estimated using GenomeScope2, which applies a polyploid-aware model (Ranallo-Benavidez et al., 2020).

To generate a high-quality, contiguous genome assembly, we applied third-generation long-read sequencing technologies: PacBio HiFi and Oxford Nanopore Technology (ONT). For HiFi sequencing, high-molecular-weight DNA was extracted from ~2g of fresh leaves using the Nanobind Plant Nuclei Kit. A circular consensus sequencing (CCS) library was prepared using the PCR-free HiFi SMRTbell Prep Kit and sequenced on the PacBio Sequel IIe system. HiFi reads (Q > 20) were derived from consensus sequences of multiple subreads.

To further improve assembly continuity and accuracy, ONT ultra-long reads were generated using the PromethION platform (Nextomics, Wuhan, China). Genomic DNA was extracted using the QIAGEN Plant Genome Kit and processed with the Ligation Sequencing Kit and Native Barcoding Expansion Kit. Sequencing was performed on an R9.4.1 flow cell according to the manufacturer’s protocol.

For chromosome-level scaffolding, a Hi-C library was constructed following a modified version of the protocol by (Belton et al., 2012). Leaf tissue was ground in liquid nitrogen and cross-linked with 4% formaldehyde. The sample was then quenched, and digested with DpnII. Biotin-14-dCTP was used to label DNA ends, followed by blunt-end ligation. Biotin from unligated ends was removed with T4 DNA polymerase. Biotin-labeled fragments were enriched with streptavidin C1 beads, A-tailed, and ligated to Illumina paired-end adapters. Hi-C libraries were sequenced on the Illumina HiSeq X Ten platform using PE150 mode.

Haplotype-aware Genome Assembly

We performed hybrid assembly (-ul) using Hifiasm v0.19.9 (Cheng et al., 2021), integrating ONT ultra-long reads and HiFi full-length reads together with Hi-C data in a single run to generate phased contigs and produce haplotype-resolved assemblies. The resulting phased assembly graph was further inspected using Bandage (Wick et al., 2015), and putative heterozygous regions were manually identified and disentangled. These two haploid contig sets after phasing assemble to yield results with approximate total length. To obtain pseudo-chromosomes of each *S. macrobotrys* haplotype, the Hi-C reads were mapped to large contigs (>1000 bp) using BWA-MEM (Li, 2013). We identified and concatenated contigs with strongly linked signals using Juice and a 3D-DNA pipeline (Dudchenko et al., 2017) to produce chromosome-level scaffolds. The final assembly with the Hi-C contact map was manually visualized and adjusted in Juicebox v1.11.08 (Durand et al., 2016). Then scaffolds were ordered and assigned to 14 pseudo-chromosomes. The remaining gaps within the pseudo-chromosomes were filled using Gapfiller v1.11 (Boetzer and Pirovano, 2012). To identify the telomeres on both ends of each pseudo-chromosome, we searched Fabales-specific telomere motifs using TIDK v0.2.31 (https://github.com/tolkit/telomeric-identifier). Pericentromeric regions and repeat units were recognized using Centromics v0.3 (https://github.com/zhangrengang/Centromics).

To assess the quality of the assembly, HiFi reads and Illumina paired reads were independently mapped to the final assembly of both haplotypes of *S. macrobotrys* genome using the parameters “-ax map-hifi” and “-ax sr” using Minimap2 v2.26 (Li, 2018). Three metrics mapping rate, genome coverage and sequencing depth were calculated using the Samtools v1.18 (Li et al., 2009). We further used BUSCO score to evaluate the completeness of the assembly within the BUSCO package v5.4.3 (Manni et al., 2021). We compared the BUSCO scores among *S. macrobotrys* and fourteen high-quality Leguminosae genomes using single-copy orthologs datasets pre-defined from three lineages Viridiplantae, Embryophyta, and Eudicot (eukaryota_obd10, 2020-09-10). We also used Merqury v1.3 (Rhie et al., 2020) to assess the *S. macrobotrys* genomes by estimating an assembly consensus quality value (QV).

Characterization for Repeats

To annotate the repeat contents in *S. macrobotrys* haplotype genomes, the transposable element (TE) annotation pipeline EarlGrey v4.4.5 (Baril et al., 2024) was employed. EarlGrey generated a non-redundant transposable element (TE) library using RepeatModeler (Flynn et al., 2020), and subsequently utilized RepeatMasker (Chen, 2004) to obtain TE annotation results for the *S. macrobotrys* genomes based on this TE library. Meanwhile, EarlGrey used LTR_Finder (Xu and Wang, 2007) to identify full-length LTR retrotransposons with intact structures. The TE annotations obtained from the final RepeatMasker run were defragmented using the loose merging process (“-loose”) in RepeatCraft (Wong and Simakov, 2019) and combined with the results from LTR_Finder. Finally, a high-quality nonredundant TE library was generated by filtering out putative spurious TE annotations (<100bp).

A TE landscape plot, which illustrates the Kimura two-parameter (K2P) distance between each identified TE and its respective consensus sequence and is broadly indicative of patterns of TE activity (recently active TE copies are assumed to have low levels of distance to their respective consensus). For LTR divergence time analysis, a substitution rate twice as high (5.38 × 10⁻⁹, see methods below) was used, based on the assumption that non-coding regions evolve faster than coding sequences (Vitte et al., 2004).

Gene Prediction and Functional Annotation

Transcriptome sequencings were conducted using 16 tissue samples including leaf, root, stem and four stage flowers (marked as LF, RT, ST and FS). For transcriptome sequencing, three pair-end RNA libraries (2×150 bp) were prepared according to the TruSeq RNA Library Preparation Kit instructions and sequenced on the Illumina Novaseq 6000 platform, yielding ~7 Gb of RNA-seq data for the leaf, flower and root samples, respectively (**Table S1**).

To mask repetitive elements for structure gene prediction, we conducted de novo repeat prediction by running RepeatModeler v2.0.3 with the default parameters, and the resulting repeat library was merged with the repeat database of Viridiplantae clade extracted from RepBase library v20181026 (Bao et al., 2015). We used this concatenated library to identify the repeat sequences of the *S. macrobotrys* genomes using RepeatMasker v4.1.2 with default parameters and produced a soft-masked genome.

Coding gene prediction was performed with multiple evidence, including ab initio searching, homology-based prediction, and transcriptome sequencing. We combined four transcriptome sequencing libraries from the roots to flower buds and mapped them to the soft-masked *S. macrobotrys* haplotype genomes using Hisat2 (Kim et al., 2019). We conducted two independent runs with BRAKER2 (Hoff et al., 2019) in RNA-seq pipeline mode and protein pipeline mode. First, RNA-seq spliced alignments were used to train the gene prediction software GeneMark-ET (Bruna et al., 2020) and AUGUSTUS (Hoff *et al.*, 2019). Second, we appended the protein sequences from seven high-quality genomes of legumes to the OrthoDB v10 library. Then, the extended OrthoDB library was used to predict gene structures under the BRAKER2 protein mode. Finally, both evidences were merged by TSEBRA (Gabriel et al., 2021) to produce the final annotations.

We employed SUPPA v2.3 (Trincado et al., 2018) to examine alternative splicing events based on the gene structure annotations with isoforms. These events were classified into seven types, including skipping exon, alternative 3′ splice sites, alternative 5′ splice sites, mutually exclusive exons, retained intron, alternative first exons, and alternative last exons. Noncoding RNA, including tRNA, rRNA, miRNA, snRNA, and snoRNA, were identified using the Infernal v1.1.4 (Nawrocki and Eddy, 2013) software with the Rfam 14 database (Kalvari et al., 2021). The ribosome DNA prediction (5S, 5.8S, 18S, and 28S rDNA) was also performed using Barrnap v0.9 (https://github.com/tseemann/barrnap).

Functional annotations for protein-coding genes were performed via four online softwares, including eggNOG-mapper v2.1.9 (Cantalapiedra et al., 2021) with precomputed eggNOG v5.0 clusters (Huerta-Cepas et al., 2019), PANNZER2 (Toronen et al., 2018), and Mercator4 v5.0 (Bolger et al., 2021). We also used the standalone annotation software InterProScan v5.62-94.0 (Jones et al., 2014) to assign proteins to the InterPro ID. We integrated these four results to obtain the final gene functional annotations with the corresponding Gene Ontology (GO) (Consortium, 2004) and Kyoto Encyclopedia of Genes and Genomes (KEGG) (Kanehisa et al., 2017) items. Transcription factors and protein kinases that regulate the activity of gene expression were identified using iTAK (Zheng et al., 2016).

Comparative genomics and Evolutionary Analysis

Genomic sequences of 44 Fabaceae species were retrieved from the Genome Database of LIS, Phytozome, Peanutbase, GigaDB and NCBI (**Table S18**). To identify gene families of all Fabaceae species, we performed reciprocal BLAST searches among the longest isoform of protein-coding genes in Orthofinder v2.5.2 (Emms and Kelly, 2019) with default inflation parameter. Lineage shared and species-specific genes were recognized. GO and KEGG enrichment analyses were conducted using the ShinyGO v0.77 (Ge et al., 2020), and a P-value <0.05 was used as the significance threshold.

To further determine the phylogenetic relationships among the species, we used BUSCO to identify universal single-copy orthologs from whole genome sequences of each species, and genes with coverage of < 80% were removed. We extracted protein-coding sequences from 1,375 single-copy genes and then aligned based on their codon positions. Protein sequences were first aligned with MAFFT v7.526 (Katoh and Standley, 2013) using the L-INS-I algorithm and then transformed to codon alignments using PAL2NAL v14.1 (Suyama et al., 2006). After cleaned the alignments using the trimAL v1.5 (Capella-Gutierrez et al., 2009), a supermatrix was built by concatenating the cleaned alignments and generated three partitions based on codon positions. The maximum likelihood (ML) tree was inferred by RAxML-NG v1.0.3 (Kozlov et al., 2019) with the partitioned GTR+GAMMA model and 200 bootstrap replicates.

Due to its numerous deciduous structures and distinctive morphological traits, Fabaceae possesses a rich fossil record among angiosperms. These continuous fossil records found in the Cenozoic that emerges from the Middle to Late Paleocene period. Based on the ML tree, MCMCTree (dos Reis et al., 2016) was used to estimate the divergence times with six fossil calibration points (**Table S19**). MCMCTree runs were conducted with the following parameters: burn-in of 2,000,000 sample frequency of 10 and sample number of 4,000,000. Two runs were performed to ensure convergence of the posterior distribution. The expansion and contraction of gene families were identified according to the divergence time along lineages with CAFE v5 (Mendes et al., 2020).

Synteny Analysis and Duplication Detection

We used MUMmer v4 (Marcais et al., 2018) to perform chromosome-scale synteny analyses between two haplotype genomes of *S. macrobotrys*. Nucmer was used to align each pair of chromosomes (-g 1000), and a delta filter was used to filter the alignment blocks (-r -q -l 200). Genomic structural variations were analyzed using SyRI (Goel et al., 2019), with emphasis on large inversions exceeding 1 Mb. To validate breakpoints of these inversions, the contig-level assemblies were aligned to the *S. macrobotrys* reference genomes. Inverted repeats and segmental duplications (SDs) near the breakpoints were also detected and characterized using the SEDEF package (Numanagic et al., 2018).

We also used MCScanX (Wang et al., 2012) to detect synteny and collinearity regions among the *S. macrobotrys* and other curated genomes in the tribe Phaseoleae (**Table S18**). With the longest protein encoded by each alternative splicing gene, reciprocal blast searches were carried by MMseqs2 (Steinegger and Soding, 2017) under the maximum sensitive setting (-I 7.5). BLAST hits with an e-value less than 1e-10 and coverage of gene paired alignment greater than 50% were selected for syntenic analysis in MCScanX. The figures illustrate the collinearity of linear patterns among various species, as analyzed by JCVI v0.9.13 (https://github.com/tanghaibao/jcvi/wiki).

Whole-genome duplication (WGD) analysis was conducted by identifying collinear blocks within *S. macrobotrys* using WGDI (Sun et al., 2022). Gene pairs in these collinear regions were extracted and aligned, and their nonsynonymous (Ka) and synonymous (Ks) substitution rates were estimated using the yn00 program in PAML (Yang, 2007). To further validate the Ks-based inference of duplication events, we calculated orthologous Ks values between S. macrobotrys and representative Fabales species (Cercis canadensis, Lupinus angustifolius, Medicago truncatula) as well as the outgroup Vitis vinifera. These cross-species Ks comparisons provided a phylogenetic and temporal framework for interpreting the Ks peaks identified in S. macrobotrys.

Differential Expression Analysis

Clean reads obtained by cDNA libraries were mapped to the reference genome using the STAR splicing aligner (Dobin et al., 2013). The counts matrix was obtained by FeatureCounts (Liao et al., 2014) and then calculating the normalized CPM (Counts Per Million) values (Nishida et al., 2017; Zhou et al., 2019). We additionally calculated FPKM (Fragments Per Kilobase Million) and TPM (Transcripts Per Kilobase Million) values to provide baseline normalized expression levels. Differential expression gene (DEG) analysis was conducted using the average CPMs of three biological replicates with R package edgeR (Chen et al., 2025) among four flower development stages and between different tissues. The significant DEGs were determined based on false discovery rate (FDR) < 0.05 and |log_2_(foldchange)| > 2.

Gene Enrichment Analyses

To further interpret the results of comparative genomic, gene family evolution, genome duplication, and population history analyses, we performed functional enrichment analysis on the associated gene sets. Gene annotation was carried out using Mercator4, assigning genes to 32 hierarchical functional categories. Two categories - unannotated genes (BIN-35) and enzyme classification (BIN-50) - were excluded, and the remaining annotated genes were used as the background. Target gene sets were filtered similarly. Enrichment analysis was conducted via the Mercator4 web service (https://www.plabipd.de/mercator_main.html) using a two-sided Fisher's exact test, with significance determined at an FDR-adjusted p-value < 0.05. Categories with enrichment factors >1 were considered significantly overrepresented, while those <1 were considered underrepresented. In addition, Gene ontology (GO) and Kyoto Encyclopedia of Genes and Genomes (KEGG) pathway enrichment analyses were performed using ClusterProfiler (Xu et al., 2024).

Demographic Analysis

Whole-genome resequencing data from the Illumina paired-end library were aligned to the *S. macrobotrys* haplotype genomes using BWA-MEM, and reads with a mapping quality below 30 were discarded. Variant calling was conducted using the bcftools mpileup and call functions. The resulting alignments were analyzed with the PSMC model (Li and Durbin, 2011). For PSMC, we set a maximum of 25 iterations, 100 bootstrap replicates, with an initial theta ratio of 5. And we used the effective population size (*Ne*) pattern "4 + 25*2 + 4 + 6" for 64 intervals. The neutral mutation rate (μ) was set to 4.73×10^-9^ substitutions per site per generation, following the estimate reported for Fabaceae (De La Torre et al., 2017). The generation time was set to three years, based on our observations of the first flowering time in plants grown from seed.

Orthologous genes, which diverge concomitantly with speciation events, offer valuable insights into the evolutionary dynamics of species lineages. We selected high-confidence orthologous gene pairs based on the intersection of syntenic blocks and best reciprocal BLAST hits. Genes of each syntenic pairs between *S. macrobotrys* subgenomes A and B were extracted from the WGDI collinearity blocks. To identify the orthology relationships, only top hits were retained from the reciprocal BLASTP results using a custom Python script. Protein and coding sequences of these orthologous gene pairs were extracted and pairwise synonymous substitution rates (*Ks*) were calculated using the synonymous_calc.py script (https://github.com/tanghaibao/bio-pipeline/blob/main/synonymous_calculation/synonymous_calc.py). With synonymous substitution rates, Divergence times (*T*) of orthologous genes were estimated using the formula *T* = *Ks*/2*r*, where *r* is the average substitution rate, representing the expected number of substitutions per site per year. The value of *r* was calculated by dividing the total branch length of the maximum likelihood phylogenetic tree by the estimated divergence time from the most recent common ancestor of Phaseoleae. Genes within specified *Ks* were identified based on the time intervals of population expansion events.

Metabolome Profiling

Untargeted metabolomic analysis was performed to characterize the metabolic profile of *S. macrobotrys*. Floral tissues from developmental stages II–IV (**Tables S22–S23**) were collected, with three biological replicates for each stage. Fresh samples were flash-frozen in liquid nitrogen, ground to a fine powder, and extracted using 80% methanol containing 0.1% formic acid. Metabolite profiling was conducted through a targeted metabolomic approach by Wuhan Metware Biotechnology Co., Ltd. (Wuhan, China) ([http://www.metware.cn/](http://www.metware.cn/)).

Data acquisition was conducted in both positive and negative ion modes. Metabolite features were detected, aligned, and annotated using Compound Discoverer (v3.1, Thermo Fisher Scientific) and matched against public databases KEGG. Putative metabolite identifications were assigned based on accurate mass, retention time, and MS/MS fragmentation patterns. Significantly regulated metabolites between groups were identified based on a combination of Variable Importance in Projection (VIP) values (VIP ≥ 1) and absolute fold change (Log2FC ≥ 1.0). VIP values were derived from the results of Orthogonal Partial Least Squares Discriminant Analysis (OPLS-DA). Differentially accumulated metabolite (DAM) pairs with an absolute Pearson’s correlation coefficient (PCC) ≥ 0.8 and *P-*value ≤ 0.05 were considered strongly correlated.

Extraction and Identification of Compounds

The experimental procedure was adapted from reference (Zeng et al., 2024) with modifications. Briefly, flower samples at different developmental stages were ground in liquid nitrogen and then lyophilized. Approximately 10 mg of the resulting dry powder was accurately weighed and extracted twice with methanol, each for 20 minutes. The combined extracts were concentrated to dryness, and the residue was reconstituted in 200 μL of methanol for subsequent high-performance liquid chromatography (HPLC) analysis. Chromatographic separation was performed on an InertSustain C18 column (4.6 × 150 mm, 5 μm). The mobile phase consisted of water containing 0.1% formic acid (v/v, solvent A) and acetonitrile (solvent B). Elution was carried out using the following gradient program: 0–5 min, 5% B; 5–6 min, 5–15% B; 6–19 min, 15% B; 19–20 min, 15–95% B; 20–23 min, 95% B. The flow rate was maintained at 1 mL/min, the column temperature was set at 40°C, and detection was performed at 280 nm. Further identification was conducted using a Thermo Scientific TSQ Endura LC-ESI-MS system equipped with a UPLC Hypersil Gold column (100 × 2.1 mm, 1.9 μm; Thermo Scientific). The chromatographic conditions were as follows: column temperature, 40°C; flow rate, 0.2 mL/min; injection volume, 1 μL. Mass spectrometry parameters were set as follows: ion source temperature, 250°C; sheath gas flow, 35 arb; auxiliary gas flow, 10 arb; ion transfer tube temperature, 275°C; spray voltage in negative mode, 2.5 kV; mass scan range, m/z 200–1000.

Metabolite-transcript Correlation Analysis

To identify candidate genes involved in the biosynthesis and regulation of the anthocyanin and flavonoid biosynthetic pathways, a weighted gene co-expression network analysis (WGCNA) based on Pearson correlation coefficients (PCCs) was performed using metabolome and transcriptome data. First, genes with undetectable or low expression levels (average CPM≤1.0) were excluded. Next, genes with a coefficient of variation (CV) > 0.5 were selected for network construction using the WGCNA R package (Langfelder and Horvath, 2008). Co-expression modules were identified with the “blockwiseModules” function, using default settings except for the following parameters: soft-thresholding power = 11, TOMtype = "unsigned", mergeCutHeight = 0.25, deepSplit = 2, and minModuleSize = 30. PCCs were then calculated to evaluate associations between genes and metabolites. Significant gene–metabolite correlations were defined as those with an absolute PCC > 0.50 and *P*-value < 0.05, where positive and negative PCC values indicated significant positive and negative correlations, respectively. The resulting networks were visualized in Cytoscape (Shannon et al., 2003).

Quantitative Real-time Polymerase Chain Reaction (qRT-PCR) Analysis

Flowers at different developmental stages were collected and immediately frozen in liquid nitrogen. Total RNA was extracted using a Plant RNA Double-Column Kit (Magen, China). Then, 500 ng of total RNA was reverse-transcribed into cDNA using the StarScript Pro All-in-one RT Mix with gDNA Remover (GenStar, China). Quantitative real-time polymerase chain reaction (qRT-PCR) was performed on Real-Time PCR System Archimed X4 using ChamQ Blue Universal SYBR qPCR Master Mix (Vazyme, China). The relative expression levels of the target genes were calculated using the 2^−ΔΔCT^ method and *Smactin1* and *SmGAPDH* as reference genes. qRT-PCR was conducted with three biological replicates. The primers used are listed in the Supplementary **Table S35**.

Reference

**Bao, W., Kojima, K.K., and Kohany, O.** (2015). Repbase Update, a database of repetitive elements in eukaryotic genomes. *Mob DNA* **6**:11.

**Baril, T., Galbraith, J., and Hayward, A.** (2024). Earl Grey: a fully automated user-friendly transposable element annotation and analysis pipeline. *Mol Biol Evol* **41**:msae068.

**Belton, J.M., McCord, R.P., Gibcus, J.H., Naumova, N., Zhan, Y., and Dekker, J.** (2012). Hi-C: a comprehensive technique to capture the conformation of genomes. *Methods* **58**:268–276.

**Boetzer, M., and Pirovano, W.** (2012). Toward almost closed genomes with GapFiller. *Genome Biol* **13**:R56.

**Bolger, M., Schwacke, R., and Usadel, B.** (2021). MapMan visualization of RNA-Seq data using Mercator4 functional annotations. *Methods Mol Biol* **2354**:195–212.

**Bruna, T., Lomsadze, A., and Borodovsky, M.** (2020). GeneMark-EP+: eukaryotic gene prediction with self-training in the space of genes and proteins. *NAR Genom Bioinform* **2**:lqaa026.

**Cantalapiedra, C.P., Hernandez-Plaza, A., Letunic, I., Bork, P., and Huerta-Cepas, J.** (2021). eggNOG-mapper v2: functional annotation, orthology assignments, and domain prediction at the metagenomic scale. *Mol Biol Evol* **38**:5825–5829.

**Capella-Gutierrez, S., Silla-Martinez, J.M., and Gabaldon, T.** (2009). trimAl: a tool for automated alignment trimming in large-scale phylogenetic analyses. *Bioinformatics* **25**:1972–1973.

**Chen, N.** (2004). Using Repeat Masker to identify repetitive elements in genomic sequences. *Current protocols in bioinformatics* **5**:4–10.

**Chen, S., Zhou, Y., Chen, Y., and Gu, J.** (2018). fastp: an ultra-fast all-in-one FASTQ preprocessor. *Bioinformatics* **34**:i884–i890.

**Chen, Y., Chen, L., Lun, A.T.L., Baldoni, P.L., and Smyth, G.K.** (2025). edgeR v4: powerful differential analysis of sequencing data with expanded functionality and improved support for small counts and larger datasets. *Nucleic Acids Res* **53**:gkaf018.

**Cheng, H., Concepcion, G.T., Feng, X., Zhang, H., and Li, H.** (2021). Haplotype-resolved de novo assembly using phased assembly graphs with hifiasm. *Nat Methods* **18**:170–175.

**Consortium, G.O.** (2004). The Gene Ontology (GO) database and informatics resource. *Nucleic acids research* **32**:D258–D261.

**De La Torre, A.R., Li, Z., Van de Peer, Y., and Ingvarsson, P.K.** (2017). Contrasting rates of molecular evolution and patterns of selection among gymnosperms and flowering plants. *Mol Biol Evol* **34**:1363–1377.

**Dobin, A., Davis, C.A., Schlesinger, F., Drenkow, J., Zaleski, C., Jha, S., Batut, P., Chaisson, M., and Gingeras, T.R.** (2013). STAR: ultrafast universal RNA-seq aligner. *Bioinformatics* **29**:15–21.

**Dolezel, J., and Bartos, J.** (2005). Plant DNA flow cytometry and estimation of nuclear genome size. *Ann Bot* **95**:99–110.

**dos Reis, M., Donoghue, P.C., and Yang, Z.** (2016). Bayesian molecular clock dating of species divergences in the genomics era. *Nat Rev Genet* **17**:71–80.

**Dudchenko, O., Batra, S.S., Omer, A.D., Nyquist, S.K., Hoeger, M., Durand, N.C., Shamim, M.S., Machol, I., Lander, E.S., Aiden, A.P., et al.** (2017). De novo assembly of the *Aedes aegypti* genome using Hi-C yields chromosome-length scaffolds. *Science* **356**:92–95.

**Durand, N.C., Robinson, J.T., Shamim, M.S., Machol, I., Mesirov, J.P., Lander, E.S., and Aiden, E.L.** (2016). Juicebox provides a visualization system for Hi-C contact maps with unlimited zoom. *Cell Syst* **3**:99–101.

**Emms, D.M., and Kelly, S.** (2019). OrthoFinder: phylogenetic orthology inference for comparative genomics. *Genome Biol* **20**:238.

**Flynn, J.M., Hubley, R., Goubert, C., Rosen, J., Clark, A.G., Feschotte, C., and Smit, A.F.** (2020). RepeatModeler2 for automated genomic discovery of transposable element families. *Proc Natl Acad Sci U S A* **117**:9451–9457.

**Gabriel, L., Hoff, K.J., Bruna, T., Borodovsky, M., and Stanke, M.** (2021). TSEBRA: transcript selector for BRAKER. *BMC Bioinformatics* **22**:566.

**Ge, S.X., Jung, D., and Yao, R.** (2020). ShinyGO: a graphical gene-set enrichment tool for animals and plants. *Bioinformatics* **36**:2628–2629.

**Goel, M., Sun, H., Jiao, W.B., and Schneeberger, K.** (2019). SyRI: finding genomic rearrangements and local sequence differences from whole-genome assemblies. *Genome Biol* **20**:277.

**Hoff, K.J., Lomsadze, A., Borodovsky, M., and Stanke, M.** (2019). Whole-genome annotation with BRAKER. *Methods Mol Biol* **1962**:65–95.

**Huerta-Cepas, J., Szklarczyk, D., Heller, D., Hernandez-Plaza, A., Forslund, S.K., Cook, H., Mende, D.R., Letunic, I., Rattei, T., Jensen, L.J., et al.** (2019). eggNOG 5.0: a hierarchical, functionally and phylogenetically annotated orthology resource based on 5090 organisms and 2502 viruses. *Nucleic Acids Res* **47**:D309–D314.

**Jones, P., Binns, D., Chang, H.Y., Fraser, M., Li, W., McAnulla, C., McWilliam, H., Maslen, J., Mitchell, A., Nuka, G., et al.** (2014). InterProScan 5: genome-scale protein function classification. *Bioinformatics* **30**:1236–1240.

**Kalvari, I., Nawrocki, E.P., Ontiveros-Palacios, N., Argasinska, J., Lamkiewicz, K., Marz, M., Griffiths-Jones, S., Toffano-Nioche, C., Gautheret, D., Weinberg, Z., et al.** (2021). Rfam 14: expanded coverage of metagenomic, viral and microRNA families. *Nucleic Acids Res* **49**:D192–D200.

**Kanehisa, M., Furumichi, M., Tanabe, M., Sato, Y., and Morishima, K.** (2017). KEGG: new perspectives on genomes, pathways, diseases and drugs. *Nucleic Acids Res* **45**:D353–D361.

**Katoh, K., and Standley, D.M.** (2013). MAFFT multiple sequence alignment software version 7: improvements in performance and usability. *Mol Biol Evol* **30**:772–780.

**Kim, D., Paggi, J.M., Park, C., Bennett, C., and Salzberg, S.L.** (2019). Graph-based genome alignment and genotyping with HISAT2 and HISAT-genotype. *Nat Biotechnol* **37**:907–915.

**Kozlov, A.M., Darriba, D., Flouri, T., Morel, B., and Stamatakis, A.** (2019). RAxML-NG: a fast, scalable and user-friendly tool for maximum likelihood phylogenetic inference. *Bioinformatics* **35**:4453–4455.

**Langfelder, P., and Horvath, S.** (2008). WGCNA: an R package for weighted correlation network analysis. *BMC Bioinformatics* **9**:559.

**Li, H.** (2013). Aligning sequence reads, clone sequences and assembly contigs with BWA-MEM. *arXiv preprint arXiv:1303.3997*.

**Li, H.** (2018). Minimap2: pairwise alignment for nucleotide sequences. *Bioinformatics* **34**:3094–3100.

**Li, H., and Durbin, R.** (2011). Inference of human population history from individual whole-genome sequences. *Nature* **475**:493–496.

**Li, H., Handsaker, B., Wysoker, A., Fennell, T., Ruan, J., Homer, N., Marth, G., Abecasis, G., Durbin, R., and Genome Project Data Processing, S.** (2009). The Sequence Alignment/Map format and SAMtools. *Bioinformatics* **25**:2078–2079.

**Liao, Y., Smyth, G.K., and Shi, W.** (2014). featureCounts: an efficient general purpose program for assigning sequence reads to genomic features. *Bioinformatics* **30**:923–930.

**Manni, M., Berkeley, M.R., Seppey, M., and Zdobnov, E.M.** (2021). BUSCO: assessing genomic data quality and beyond. *Curr Protoc* **1**: e323.

**Marcais, G., and Kingsford, C.** (2011). A fast, lock-free approach for efficient parallel counting of occurrences of k-mers. *Bioinformatics* **27**:764–770.

**Marcais, G., Delcher, A.L., Phillippy, A.M., Coston, R., Salzberg, S.L., and Zimin, A.** (2018). MUMmer4: A fast and versatile genome alignment system. *PLoS Comput Biol* **14**: e1005944.

**Mendes, F.K., Vanderpool, D., Fulton, B., and Hahn, M.W.** (2020). CAFE 5 models variation in evolutionary rates among gene families. *Bioinformatics* **36**:5516–5518.

**Nawrocki, E.P., and Eddy, S.R.** (2013). Infernal 1.1: 100-fold faster RNA homology searches. *Bioinformatics* **29**:2933–2935.

**Nishida, S., Kakei, Y., Shimada, Y., and Fujiwara, T.** (2017). Genome-wide analysis of specific alterations in transcript structure and accumulation caused by nutrient deficiencies in Arabidopsis thaliana. *Plant J.* **91**:741–753.

**Numanagic, I., Gokkaya, A.S., Zhang, L., Berger, B., Alkan, C., and Hach, F.** (2018). Fast characterization of segmental duplications in genome assemblies. *Bioinformatics* **34**:i706–i714.

**Ranallo-Benavidez, T.R., Jaron, K.S., and Schatz, M.C.** (2020). GenomeScope 2.0 and Smudgeplot for reference-free profiling of polyploid genomes. *Nat Commun* **11**:1432.

**Rhie, A., Walenz, B.P., Koren, S., and Phillippy, A.M.** (2020). Merqury: reference-free quality, completeness, and phasing assessment for genome assemblies. *Genome Biol* **21**:245.

**Shannon, P., Markiel, A., Ozier, O., Baliga, N.S., Wang, J.T., Ramage, D., Amin, N., Schwikowski, B., and Ideker, T.** (2003). Cytoscape: a software environment for integrated models of biomolecular interaction networks. *Genome Research* **13**:2498–2504.

**Steinegger, M., and Soding, J.** (2017). MMseqs2 enables sensitive protein sequence searching for the analysis of massive data sets. *Nat Biotechnol* **35**:1026–1028.

**Sun, P., Jiao, B., Yang, Y., Shan, L., Li, T., Li, X., Xi, Z., Wang, X., and Liu, J.** (2022). WGDI: A user-friendly toolkit for evolutionary analyses of whole-genome duplications and ancestral karyotypes. *Mol Plant* **15**:1841–1851.

**Suyama, M., Torrents, D., and Bork, P.** (2006). PAL2NAL: robust conversion of protein sequence alignments into the corresponding codon alignments. *Nucleic Acids Res* **34**:W609–612.

**Toronen, P., Medlar, A., and Holm, L.** (2018). PANNZER2: a rapid functional annotation web server. *Nucleic Acids Res* **46**:W84–W88.

**Trincado, J.L., Entizne, J.C., Hysenaj, G., Singh, B., Skalic, M., Elliott, D.J., and Eyras, E.** (2018). SUPPA2: fast, accurate, and uncertainty-aware differential splicing analysis across multiple conditions. *Genome Biol* **19**:40.

**Vitte, C., Ishii, T., Lamy, F., Brar, D., and Panaud, O.** (2004). Genomic paleontology provides evidence for two distinct origins of Asian rice (Oryza sativa L.). *Mol Genet Genomics* **272**:504–511.

**Wang, Y., Tang, H., Debarry, J.D., Tan, X., Li, J., Wang, X., Lee, T.H., Jin, H., Marler, B., Guo, H., et al.** (2012). MCScanX: a toolkit for detection and evolutionary analysis of gene synteny and collinearity. *Nucleic Acids Res* **40**: e49.

**Wick, R.R., Schultz, M.B., Zobel, J., and Holt, K.E.** (2015). Bandage: interactive visualization of de novo genome assemblies. *Bioinformatics* **31**:3350–3352.

**Wong, W.Y., and Simakov, O.** (2019). RepeatCraft: a meta-pipeline for repetitive element de-fragmentation and annotation. *Bioinformatics* **35**:1051–1052.

**Xu, S., Hu, E., Cai, Y., Xie, Z., Luo, X., Zhan, L., Tang, W., Wang, Q., Liu, B., Wang, R., et al.** (2024). Using clusterProfiler to characterize multiomics data. *Nat Protoc* **19**:3292–3320.

**Xu, Z., and Wang, H.** (2007). LTR_FINDER: an efficient tool for the prediction of full-length LTR retrotransposons. *Nucleic Acids Res* **35**:W265–268.

**Yang, Z.** (2007). PAML 4: phylogenetic analysis by maximum likelihood. *Mol Biol Evol* **24**:1586–1591.

**Zeng, S., Wang, Z., Shi, D., Yu, F., Liu, T., Peng, T., Bi, G., Yan, J., and Wang, Y.** (2024). The high-quality genome of *Grona styracifolia* uncovers the genomic mechanism of high levels of schaftoside, a promising drug candidate for treatment of COVID-19. *Hortic. Res.* **11**: uhae089.

**Zheng, Y., Jiao, C., Sun, H., Rosli, H.G., Pombo, M.A., Zhang, P., Banf, M., Dai, X., Martin, G.B., Giovannoni, J.J., et al.** (2016). iTAK: a program for genome-wide prediction and classification of plant transcription factors, transcriptional regulators, and protein kinases. *Mol Plant* **9**:1667–1670.

**Zhou, P., Hirsch, C. N., Briggs, S. P., and Springer, N. M.** (2019). Dynamic patterns of gene expression additivity and regulatory variation throughout maize development. *Mol. Plant* **12**:410–425.
